# Tauroursodeoxycholic Acid Mitigates High Fat Diet-Induced Cardiomyocyte Contractile and Intracellular Ca^2+^ Anomalies

**DOI:** 10.1371/journal.pone.0063615

**Published:** 2013-05-07

**Authors:** Subat Turdi, Nan Hu, Jun Ren

**Affiliations:** Center for Cardiovascular Research and Alternative Medicine, Division of Pharmaceutical Sciences, University of Wyoming College of Health Sciences, Laramie, Wyoming, United States of America; Massachusetts Eye & Ear Infirmary, Harvard Medical School, United States of America

## Abstract

**Objectives:**

The endoplasmic reticulum (ER) chaperone tauroursodeoxycholic acid (TUDCA) has exhibited promises in the treatment of obesity, although its impact on obesity-induced cardiac dysfunction is unknown. This study examined the effect of TUDCA on cardiomyocyte function in high-fat diet-induced obesity.

**Methods:**

Adult mice were fed low or high fat diet for 5 months prior to treatment of TUDCA (300 mg/kg. i.p., for 15d). Intraperitoneal glucose tolerance test (IPGTT), cardiomyocyte mechanical and intracellular Ca^2+^ property, insulin signaling molecules including IRS-1, Akt, AMPK, ACC, GSK-3β, c-Jun, ERK and c-Jun N terminal kinase (JNK) as well as ER stress and intracellular Ca^2+^ regulatory proteins were examined. Myocardial ultrastructure was evaluated using transmission electron microscopy (TEM).

**Results:**

High-fat diet depressed peak shortening (PS) and maximal velocity of shortening/relengthenin as well as prolonged relengthening duration. TUDCA reversed or overtly ameliorated high fat diet-induced cardiomyocyte dysfunction including prolongation in relengthening. TUDCA alleviated high-fat diet-induced decrease in SERCA2a and phosphorylation of phospholamban, increase in ER stress (GRP78/BiP, CHOP, phosphorylation of PERK, IRE1α and eIF2α), ultrastructural changes and mitochondrial permeation pore opening. High-fat diet feeding inhibited phosphorylation of AMPK and promoted phosphorylation of GSK-3β. TUDCA prevented high fat-induced dephosphorylation of AMPK but not GSK-3β. High fat diet promoted phosphorylation of IRS-1 (Ser^307^), JNK, and ERK without affecting c-Jun phosphorylation, the effect of which with the exception of ERK phosphorylation was attenuated by TUDCA.

**Conclusions:**

These data depict that TUDCA may ameliorate high fat diet feeding-induced cardiomyocyte contractile and intracellular Ca^2+^ defects through mechanisms associated with mitochondrial integrity, AMPK, JNK and IRS-1 serine phosphorylation.

## Introduction

According to the 2011 estimate of the Center for Disease Control and Prevention (CDC), nearly 26 million Americans are afflicted with diabetes mellitus while 79 million individuals are considered prediabetic[1]. Both clinical and experimental evidence has identified a variety of predisposing factors for diabetes, with obesity being one independent causative factor for the increased prevalence of type 2 diabetes [2]. In addition, uncorrected obesity adds to the overall health burden including cardiovascular diseases, pulmonary diseases, cancer, and sleep disorders [2]–[4]. Obesity is drawing more and more attention as a pandemic health issue. Therefore, the search for effective management strategies for obesity-related cardiovascular diseases becomes a rather compelling health care initiative. However, adequate management against obesity-related cardiovascular diseases has been somewhat dismal due to the complex and multifactorial etiology of obesity-induced anomalies. Among the array of pathophysiological machineries identified for obesity pathology such as genetics, inflammation, insulin resistance, oxidative stress, autophagy and apoptosis, enhanced endoplasmic reticulum (ER) stress has emerged as a crucial factor in obesity-induced metabolic derangement [5]. The ER is usually responsible for the synthesis and folding of membrane and secretory proteins while perturbation of such process triggers ER stress leading to the onset and development of a wide variety of diseases [6]. Ample evidence has depicted a pivotal role of ER stress in the pathogenesis of insulin resistance, alcoholism, diabetes and obesity [5], [7]–[9]. In particular, ER stress is known to suppress insulin receptor signaling via hyperactivation of c-Jun N-terminal kinase (JNK) and subsequently serine phosphorylation of insulin receptor substrate-1 (IRS-1), suggesting an essential role of ER stress in the etiology of insulin resistance and type 2 diabetes [5]. More recent evidence also revealed a pivotal role of ER stress in the pathogenesis of heart problems [10], [11]. For example, pressure overload was shown to result in sustained ER stress, leading to cardiomyocyte apoptosis during transition from cardiac hypertrophy to heart failure [12]. ER stress is known to be associated with cardiomyocyte apoptosis [13], [14] and deteriorated ischemia/reperfusion [15], [16]. Not surprisingly, ER stress may be considered as a therapeutic target for a number of cardioprotective signaling molecules including AMP-activated protein kinase (AMPK) [17]. AMPK has been demonstrated to retard ER stress through eEF2 inactivation, resulting in preservation of sarco(endo)plasmic reticulum (SERCA) function and intracellular Ca^2+^ homeostasis [17], [18].

Recent evidence has revealed that ursodeoxycholic acid (UDCA), an endogenous bile acid, and its taurine-conjugated derivative tauroursodeoxycholic acid (TUDCA) possess potent regulatory effects on ER stress in obesity and diabetes [5], [9], [19], [20]. UDCA exists in human bile at low concentrations (3% of bile acids) and has been used in Chinese medicine for centuries for a variety of biliary and liver diseases. Since the mid-1980s, UDCA has been used clinically for liver diseases in the Western world [21]. UDCA and TUDCA are capable of inhibiting ER stress-induced apoptosis *in vivo* and *in vitro* [9], [21]–[23]. For example, TUDCA administration to obese and diabetic mice has been shown to normalize hyperglycemia, insulin sensitivity, resolve fatty liver disease, and enhance insulin action in liver, muscle and adipose tissues [9]. To this end, the present study was designed to examine the impact of ER stress correction using TUDCA in high fat diet-induced changes in cardiac contractile function, intracellular Ca^2+^ homeostasis and ultrastructural properties as well as the underlying mechanisms involved.

## Materials and Methods

### Experimental procedures

All animal procedures were approved by the Institutional Animal Use and Care Committee at the University of Wyoming (Laramie, WY). In brief, 3–4 month-old male C57BL/6 mice were fed either a normal low fat (LF, 10 and 70% of total calorie from fat and carbohydrate, respectively; Research Diets Inc., New Brunswick, NJ, Catalogue #D12450B) or a high-fat [HF, 45% of total calorie from fat (consisting of 37% from saturated fat, 46% and 19% from mono- and polyunsaturated fatty acids, respectively) and 35% of total calorie from carbohydrate; Research Diets Inc., Catalogue #D12451] for 5 months. The high-fat diet was calorically rich (4.83 kcal/g vs. 3.91 kcal/g in low-fat diet) due to the higher fat composition [24], which is also referred to as the “Western diet” [25]. Body weights and food intake were assessed weekly. After 5 months of feeding, animals were randomly divided into 4 groups to assign either low or high fat diet receiving a 300 g/kg/d.i.p., TUDCA (Gibbstown, NJ; Catalogue #580549) in 0.1% carboxymethylcellulose (CMC) or vehicle (0.1% CMC only) for a period of 15 days [9], [26]. Mice were housed in clear plastic cages in a temperature- and humidity-controlled environment with a 12/12 light/dark cycle and allowed access to their designated diets and water *ad libitum*.

### Intraperitoneal glucose tolerance test (IPGTT)

After diet feeding and TUDCA treatment period, mice were fasted for 12 hrs and were given an intraperitoneal injection of glucose (2 g/kg b.w.). Blood samples were drawn from tail veins and glucose levels were determined immediately before glucose challenge, as well as 15, 60 and 120 min thereafter using an Accu-Chek III glucose analyzer (Boehringer Mannheim Diagnostics, Indianapolis, IN) [27].

### Isolation of murine cardiomyocytes

Hearts were rapidly removed from anesthetized (ketamine 80 mg/kg and xylazine 12 mg/kg, i.p.) mice and mounted onto a temperature-controlled (37°C) Langendorff system. After perfusing with a modified Tyrode's solution (Ca^2+^ free) for 2 min, hearts were digested with a Ca^2+^-free KHB buffer containing liberase blendzyme 4 (Hoffmann-La Roche Inc., Indianapolis, IN) for 20 min. The modified Tyrode solution (pH 7.4) contained the following (in mM): NaCl 135, KCl 4.0, MgCl_2_ 1.0, HEPES 10, NaH_2_PO_4_ 0.33, glucose 10, butanedione monoxime 10, and was gassed with 5% CO_2_–95% O_2_. The digested hearts were then removed from the cannula and left ventricle was cut into small pieces. Tissue pieces were gently agitated and pellet of cells was resuspended. Extracellular Ca^2+^ was added incrementally back to 1.20 mM over a period of 30 min. Isolated cardiomyocytes were used for experiments within 8 hrs of isolation. Normally, a yield of ∼50–60% viable rod-shaped cardiomyocytes with clear sarcomere striations was achieved. Cardiomyocytes with obvious sarcolemmal blebs or spontaneous contractions were not chosen for study. Only rod-shaped cardiomyocytes with clear edges were selected for mechanical and intracellular Ca^2+^ studies [24].

### Cell shortening/relengthening

Mechanical properties of cardiomyocytes were assessed using a SoftEdge MyoCam® system (IonOptix Corporation, Milton, MA) [24]. In brief, cardiomyocytes were placed in a temperature-controlled Warner chamber mounted onto the stage of an inverted microscope (Model IX-70, Olympus Incorporation, Tokyo, Japan) and superfused (∼1 ml/min at 25°C) with a contractile buffer containing (in mM): 131 NaCl, 4 KCl, 1 CaCl_2_, 1 MgCl_2_, 10 glucose, 10 HEPES, at pH 7.4. Cardiomyocytes were field stimulated with a supra-threshold voltage at a frequency of 0.5 Hz (unless otherwise indicated), 3 msec duration, using a pair of platinum wires connected to a FHC stimulator (Brunswick, NE). The myocyte being studied was displayed on the computer monitor using an IonOptix MyoCam camera. An IonOptix SoftEdge software was used to capture changes in cell length during shortening and relengthening. Cell shortening and relengthening were assessed using the following indices: peak shortening (PS) - indicative of peak ventricular contractility, time-to-PS (TPS) - indicative of contraction duration, and time-to-90% relengthening (TR_90_) - represents cardiomyocyte relaxation duration, maximal velocities of shortening (+dL/dt) and relengthening (− dL/dt) - indicatives of maximal velocities of ventricular pressure rise/fall. In the case of altered stimulus frequency from 0.1 to 5.0 Hz, the steady state contraction was achieved (after the first 5–6 beats) before PS was recorded at 37°C.

### Intracellular Ca^2+^ transient measurement

Myocytes were loaded with fura-2/AM (0.5 µM) for 10 min and fluorescence measurements were recorded at 25°C using a dual-excitation fluorescence photomultiplier system (Ionoptix). Myocytes were placed on an Olympus IX-70 inverted microscope and imaged through a Fluor ×40 oil objective. Cells were exposed to light emitted by a 75 W lamp and passed through either a 360 or a 380 nm filter, while being stimulated to contract at 0.5 Hz. Fluorescence emissions were detected between 480 and 520 nm by a photomultiplier tube after first illuminating the cells at 360 nm for 0.5 sec then at 380 nm for the duration of the recording protocol (333 Hz sampling rate). The 360 nm excitation scan was repeated at the end of the protocol and qualitative changes in intracellular Ca^2+^ concentration were inferred from the ratio of fura-2 fluorescence intensity (FFI) at two wavelengths (360/380). Steady state intracellular Ca^2+^ transients (2 traces from each cell) were used. Fluorescence decay time was measured as an indication of the intracellular Ca^2+^ clearing rate. intracellular Ca^2+^ transient decay time constant calculated using a single exponential equation Single exponential curve fit programs were applied to calculate the intracellular Ca^2+^ decay constant [24].

### Transmission electron microscopy (TEM)

Left ventricle was fixed with 2.5% glutaraldehyde/1.2% acrolein in a fixative buffer (0.1 M cacodylate, 0.1 M sucrose, pH 7.4) and 1% osmium tetroxide, followed by treatment with 1% uranyl acetate. The samples were dehydrated through a graded series of ethanol concentrations before being embedded in LX112 resin (LADD Research Industries, Burlington, VT). Ultra-thin sections (∼50 nm) were cut on ultramicrotome, stained with uranyl acetate, followed by lead citrate, and visualized using a Hitachi H-7000 Transmission Electron Microscope equipped with a 4K×4K cooled CCO digital camera [28]. Quantitative analysis of electron microscopy (magnification ×20000) of >10 randomly selected images from each mouse group were analyzed in a blinded fashion using the NIH ImageJ software. At least 30 mitochondria were randomly selected per animal group and their minimum and maximum gray values were determined. Mitochondrial abnormalities were expressed as the difference between minimum and maximum gray values [29].

### NAD^+^ measurements

The NAD levels from ventricular tissues were measured as described [30] with minor modifications. In brief, 30 mg of frozen tissue was crushed and suspended with 180 µl of 0.6 M perchloric acid. The mixture was then homogenized and centrifuged at 13,000 *g* for 5 min. After neutralization with 3 M potassium hydroxide, NAD^+^ concentrations were determined fluorometrically using alcohol dehydrogenase activity. Excitation was at 339 nm and emission wavelength at 460 nm monitored using a SpectraMax Gemini fluorescent microplate reader.

### Western blot analysis

Expression of the intracellular Ca^2+^ regulatory proteins [SERCA2a, Na^+^-Ca^2+^ exchanger, phospholamban (PLB)], the ER stress markers [GRP78/Bip, phospho-eIF2α (peIF2α), PERK and CHOP], the major glucose and insulin regulatory proteins including Akt, phospho-Akt (pAkt, Ser^473^), AMPKα, pAMPK (Thr^172^), ACC, pACC (Ser^79^), GSK-3β, pGSK-3β (Ser^9^), IRS-1, pIRS-1 (Ser^307^), cJun, pcJun, JNK, pJNK, ERK and pERK were examined using Western blot analysis. Samples containing equal amount of proteins (50 µg) were separated on 10% SDS-polyacrylamide gels using a minigel apparatus (Mini-PROTEAN II, Bio-Rad Laboratories, Hercules, CA) and were transferred to nitrocellulose membranes. Membranes were incubated overnight at 4°C with anti-PERK (1∶1,000), anti-pPERK (1∶500), anti-pIRE (1∶1,000), anti-CHOP (1∶1,000), anti-eIF2α (1∶500), anti-peIF2α (1∶500), anti-SERCA2a (1∶1,000), anti-Na^+^-Ca^2+^ exchanger (1∶1,000), anti-PLB (1∶1,000), anti-pPLB (Ser^16^, 1∶1,000), anti-IRS-1 (1∶1,000), anti-pIRS-1 (1∶1000), anti-Akt (1∶1,000), anti-pAkt (1∶1,000), anti-AMPKα (1∶1,000), anti-pAMPK (1∶1,000), anti-ACC (1∶1,000), anti-pACC (1∶1,000), anti-GSK-3β (1∶1,000), anti-pGSK-3β (1∶1,000), anti-c-Jun (1∶1,000), anti-pc-Jun (1∶1,000), anti-JNK (1∶1,000), anti-pJNK (1∶1,000), anti-ERK (1∶1,000), and anti-pERK (1∶1,000) antibodies. Anti-SERCA2a was purchased from Affinity BioReagents (Golden, CO). Anti-pPLB antibody was purchased from Abcam (Cambridge, MA). All other antibodies were obtained from Cell Signaling (Beverly, MA). After incubation with the primary antibody, blots were incubated with an anti-rabbit IgG HRP-linked antibody at a dilution of 1∶5,000 for 1 hr at room temperature. The intensity of bands was measured with a scanning densitometer (model GS-800; Bio-Rad). α-tubulin was used as the internal loading control [24].

### Statistical analysis

Data were Mean±SEM. Statistical significance (p<0.05) for each variable was determined by a 2-factor ANOVA followed by a Bonferroni *post hoc* test.

## Results

### General feature of mice and IPGTT

**Figure 1:**
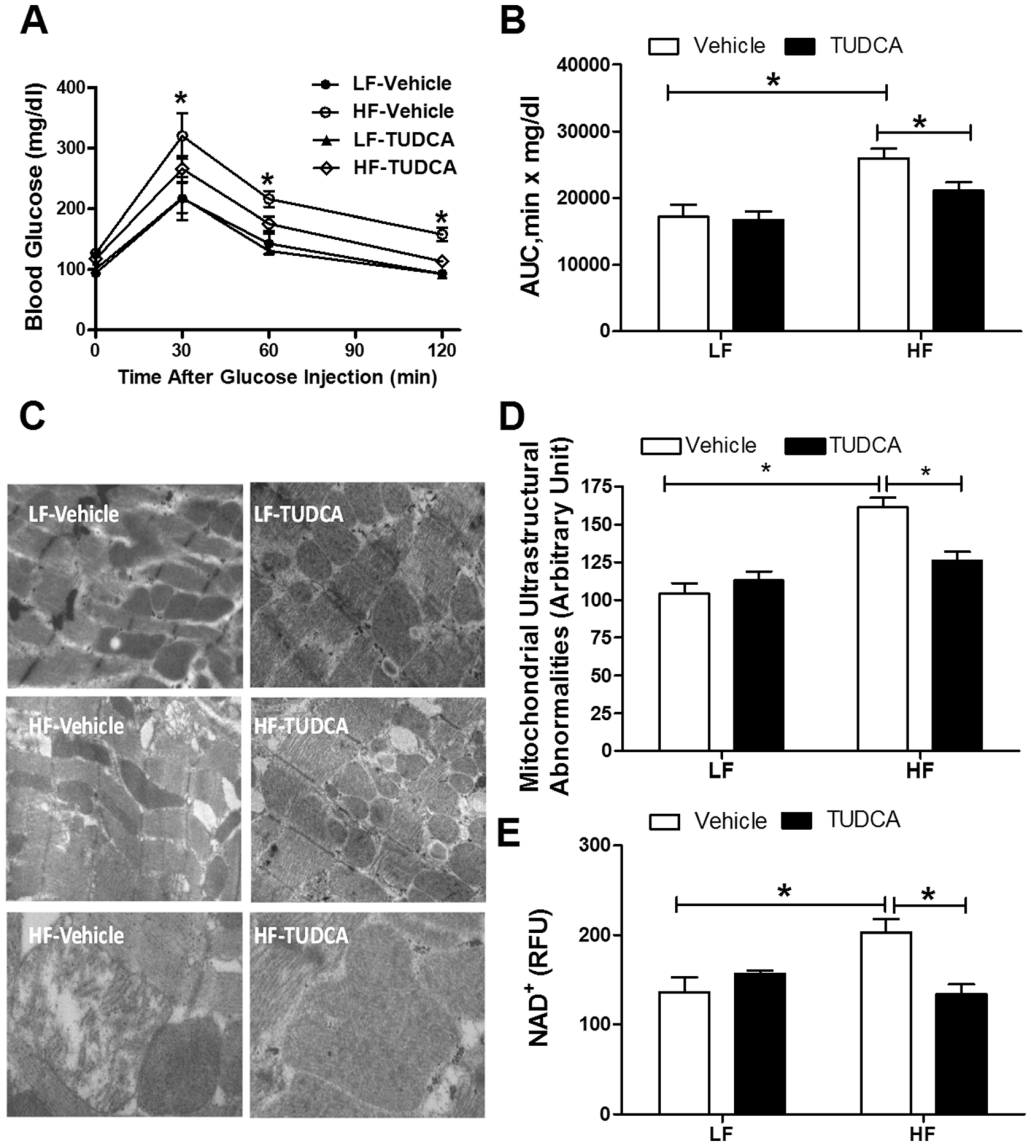
Intraperitoneal glucose tolerance test (IPGTT, 2 g/kg), myocardial ultrastructural and mitochondrial properties in low fat (LF) or high fat (HF)-fed C57 mice with or without TUDCA treatment (300 mg/kg for 15 days). A: Serum glucose levels following intraperitoneal glucose challenge; B: Area under the curve (AUC) for IPGTT; C: Transmission electron microscopic micrographs of left ventricular tissue; Normal myofilament and mitochondrial ultrastructure may be seen in LF, LF-TUDCA and HF-TUDCA groups while myocardium in HF group displays irregular and deformed myofibril structure, some swollen mitochondria with cristae loss. Original magnification  = ×15,000 for upper and middle panels; Original magnification  = ×30,000 for bottom panels; D: Quantitative analyses of mitochondrial ultrastructural abnormalities and E: Mitochondrial permeation transition pore (mPTP) opening measured using NAD^+^ level; Mean±SEM, n = 4 mice per group, *p<0.05.

As expected, high fat diet significantly increased body weight, the effect of which was unaffected by TUDCA treatment (LF: 27.9±0.6 g; HF: 33.0±1.1 g; LF-TUDCA: 27.6±0.9 g; HF-TUDCA: 33.7±1.5 g; p<0.05 between any LF and HF groups, n = 5–7 mice per group). Similarly, high fat diet feeding overtly elevated heart weight, the effect of which was unaffected by TUDCA treatment (LF: 134±6 mg; HF: 189±9 mg; LF-TUDCA: 143±9 mg; HF-TUDCA: 194±9 mg; p<0.05 between any LF and HF groups, n = 5–7 mice per group). Given that high fat intake is one of the risk factors for insulin resistance [28], [31], intraperitoneal glucose tolerance test was performed at the end of the 15-day TUDCA or vehicle treatment period. Neither high fat diet feeding nor TUDCA treatment significantly affected basal blood glucose levels. Following acute intraperitoneal glucose challenge, blood glucose levels in low fat diet-fed mice started to decline after peaking at around 30 min, and returned to nearly baseline after 120 min. However, the post-challenge glucose levels maintained at much high levels from 30 to 120 min in high fat diet-fed mice, indicating glucose intolerance. TUDCA treatment significantly facilitated glucose clearance rate and decreased the area underneath the IPGTT curve in high fat diet-fed mice without affecting those in low fat diet-fed mice, indicating a beneficial effect of ER stress inhibition on glucose metabolism (Fig. 1A-B).

### Electron microscopy and mitochondrial permeation transition pore (mPTP) opening

TEM examination revealed that high fat diet feeding triggered extensive focal damage in myocardial tissue, as evidenced by disorganized myofibrils, swelling vacuolization, and in some cases, loss of cristae of mitochondria. Quantitative analysis of TEM images (evaluated by the difference between minimum and maximum gray values) [29] revealed that TUDCA treatment effectively alleviated high fat diet-induced ultrastructural abnormalities without eliciting any notable effect itself (Fig. 1C-D). In line with the mitochondrial ultrastructural observation, myocardial NAD^+^ release, an indicative for mPTP opening, was significantly elevated in high fat diet-fed mouse hearts, the effect of which was reversed by TUDCA treatment. TUDCA treatment did not affect mPTP opening in low fat diet group (Fig. 1E).

### Mechanical and intracellular Ca^2+^ properties of cardiomyocytes

**Figure 2:**
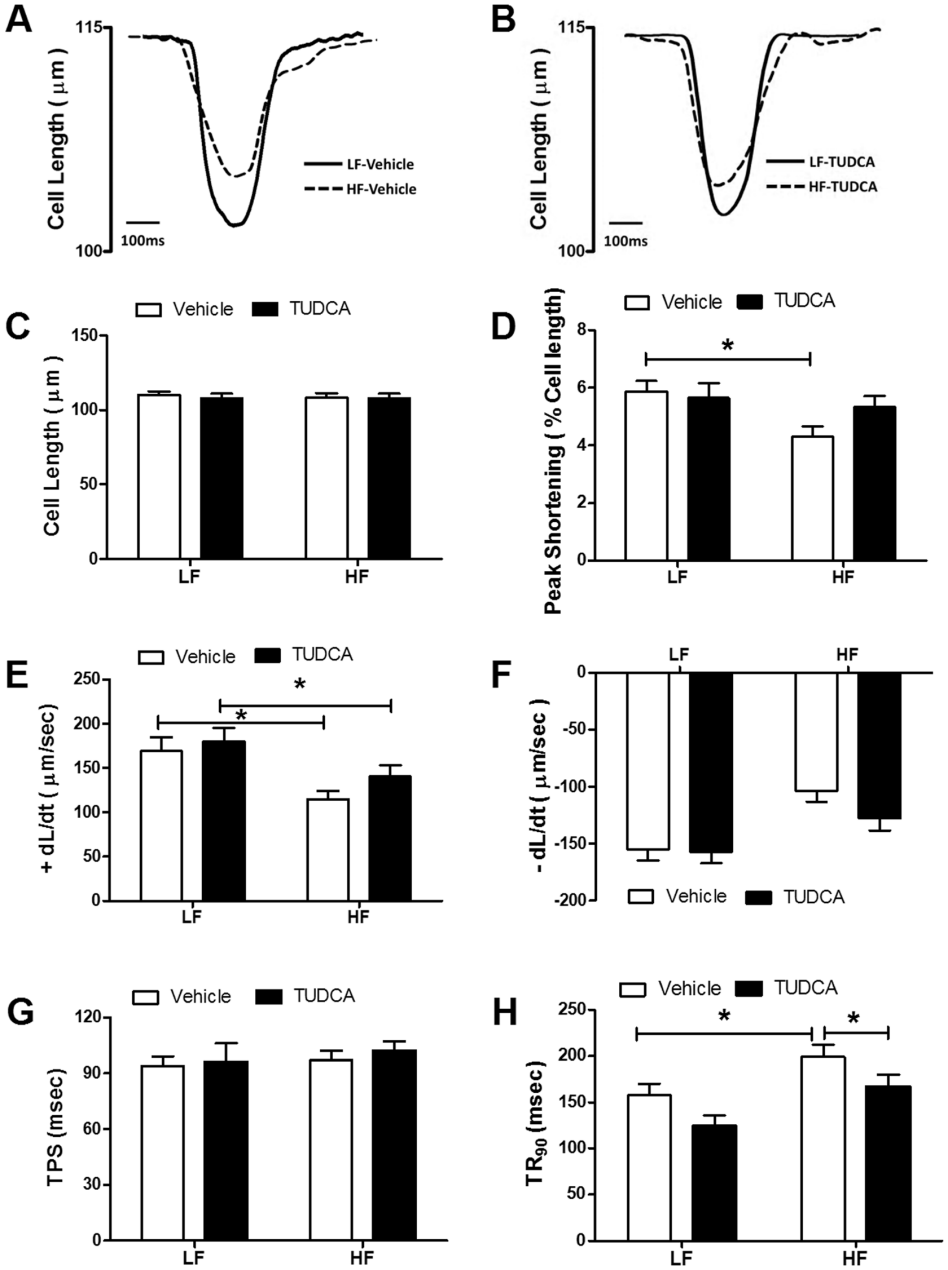
Cardiomyocyte contractile function at 25°C in low fat (LF) or high fat (HF)-fed C57 mice with or without TUDCA treatment (300 mg/kg for 15 days). A: Representative traces depicting cell shortening in LF and HF-fed mice; B: Representative traces depicting cell shortening in LF and HF-fed mice with TUDCA treatment; C: Resting cell length; D: Peak shortening (PS, normalized to resting cell length); E: Maximal velocity of shortening (+dL/dt); F: Maximal velocity of relengthening (−dL/dt); G: Time-to-PS (TPS); and I: Time-to-90% relengthening (TR_90_). Mean±SEM, n = 60–64 cells from 3 mice per group, *p<0.05 (two-way ANOVA).

**Figure 3:**
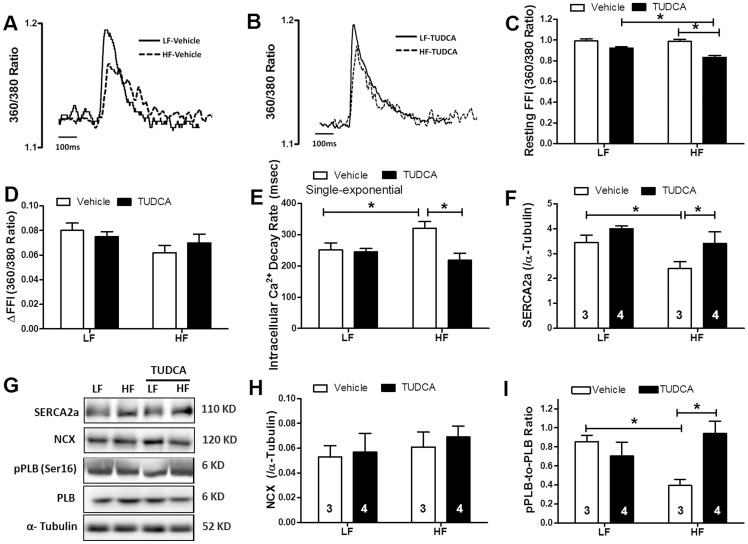
Intracellular Ca^2+^ and intracellular Ca^2+^ regulatory protein properties in hearts from low fat (LF) or high fat (HF)-fed C57 mice with or without TUDCA treatment (300 mg/kg for 15 days). A: Representative traces depicting intracellular Ca^2+^ transients in LF and HF-fed mice; B: Representative traces depicting Ca^2+^ transients in LF and HF-fed mice with TUDCA treatment; C: Baseline intracellular Ca^2+^ levels; D: Rise in intracellular Ca^2+^ in response to electrical stimulus shown as changes in Fura-2 fluorescence intensity (ΔFFI); E: Single exponential intracellular Ca^2+^ decay rate; F: Representative gel blots depicting levels of SERCA2a, Na^+^-Ca^2+^ exchanger (NCX), total/phosphorylated phospholamban (pPLB) and α-tubulin (loading control) using specific antibodies; G: SERCA2a expression; H: NCX expression; and I: pPLB (Ser^16^)-to-PLB ratio. Mean±SEM, n = 44–47 cells from 3 mice (panels A-E); or denoted in the graphs (panels F-I); *p<0.05 (two-way ANOVA).

Cardiomyocyte mechanical assessment displayed comparable resting cell length in low or high fat diet-fed group. However at a stimulus frequency of 0.5 Hz and a recording temperature of 25°C, high fat diet feeding significantly depressed cardiomyocyte peak shortening amplitude and maximal velocity of shortening/relengthening (±dL/dt) and prolonged duration of relengthening (TR_90_) associated with unchanged shortening duration (TPS) compared with low fat diet-fed mice. High fat diet-induced cardiomyocyte contractile anomalies were significantly attenuated or mitigated by TUDCA. TUDCA failed to elicit any notable effect on cardiomyocyte mechanics (Fig. 2). Given that rodent hearts beat physiologically at relatively higher frequencies (∼300–400 bpm), peak shortening amplitude was measured at 37°C in murine cardiomyocytes at increased stimulus frequencies (0.1 – 5.0 Hz). Our data shown in Table 1 revealed that high fat diet-induced cardiomyocyte contractile anomalies seen at 0.5 Hz at 25°C were also notable between 0.1 and 5.0 Hz at 37°C (with the exception of TR_90_ at 5.0 Hz). While TUDCA treatment did not affect cardiomyocyte mechanical properties between 0.1 and 5.0 Hz, it significantly attenuated or mitigated high fat diet feeding-induced contractile dysfunction. Neither high fat diet nor TUDCA significantly affected TPS at the stimulus frequencies tested at 37°C (0.1–5.0 Hz, data not shown). To explore the possible underlying mechanism(s) behind TUDCA-induced benefit against high fat diet-induced cardiomyocyte mechanical defect, intracellular Ca^2+^ handling was assessed at 25°C using Fura-2 fluorescence microscopy. As shown in Fig. 3A-E, cardiomyocytes from high fat diet-fed mice displayed elevated resting fura-2 fluorescence intensity (FFI), decreased rise of FFI in response to electrical stimuli (ΔFFI) and prolonged intracellular Ca^2+^ decay, the effects of which were significantly alleviated or ablated by TUDCA. TUDCA did not alter intracellular Ca^2+^ properties in cardiomyocytes from low fat-fed mice.

**Table 1:** Cardiomyocyte contractile function at stimulus frequencies of 0.1–5 Hz (at 37°C) in low fat (LF) or high fat (HF)-fed C57 mice with or without TUDCA treatment (300 mg/kg, 15 d).

Peak shortening	Vehicle-LF	TUDCA-LF	Vehicle-HF	TUDCA-HF
0.1 Hz	6.41±0.72	6.11±0.82	5.07±0.49*	5.97±0.53
0.5 Hz	4.98±0.71	4.48±0.57	3.75±0.37*	4.64±0.48
1.0 Hz	3.58±0.63	3.41±0.46	2.70±0.37*	3.34±0.28
3.0 Hz	3.42±0.75	3.12±0.58	2.26±0.36*	3.08±0.29
5.0 Hz	3.30±0.70	2.99±0.56	1.97±0.30*	2.83±0.26
**+dL/dt ( µm/sec)**	**Vehicle-LF**	**TUDCA-LF**	**Vehicle-HF**	**TUDCA-HF**
0.1 Hz	152.5±19.6	153.0±16.7	113.5±14.1*	145.8±15.1
0.5 Hz	120.0±20.1	116.4±13.9	92.1±10.6*	108.0±11.8
1.0 Hz	87.2±18.4	90.5±11.4	65.9±9.2*	83.1±8.6
3.0 Hz	72.7±13.7	78.2±12.7	50.7±7.4*	75.2±9.2
5.0 Hz	58.0±11.1	66.8±9.1	38.7±9.1*	60.9±6.9
**−dL/dt ( µm/sec)**	**Vehicle-LF**	**TUDCA-LF**	**Vehicle-HF**	**TUDCA-HF**
0.1 Hz	−142.9±19.2	−135.5±15.8	−111.5±14.7*	−131.3±13.0
0.5 Hz	−112.6±16.9	−118.6±16.4	−91.2±12.4*	−113.3±17.0
1.0 Hz	−75.5±13.7	−89.2±14.1	−55.7±9.0*	−70.5±9.4
3.0 Hz	−67.1±13.8	−65.9±14.9	−40.6±7.2*	−62.2±8.4
5.0 Hz	−55.1±10.7	−56.5±11.2	−35.1±5.8*	−52.3±6.6
**TR_90_ (msec)**	**Vehicle-LF**	**TUDCA-LF**	**Vehicle-HF**	**TUDCA-HF**
0.1 Hz	171.1±11.3	175.3±17.4	215.6±18.7*	176.7±15.9
0.5 Hz	170.2±12.7	165.9±14.2	224.5±14.1*	175.8±16.4
1.0 Hz	172.5±20.3	170.6±20.0	219.0±17.3*	173.4±17.1
3.0 Hz	98.8±8.2	113.3±7.8	126.7±10.7*	103.1±7.9
5.0 Hz	96.5±7.9	96.9±9.29	102.2±7.8	94.2±10.3

Peak shortening is presented as% change of resting cell length;±dL/dt: Maximal velocity of shortening/relengthening; TR_90_: Time-to-90% relengthening; Mean±SEM, n = 32–33 cells from 4 mice per group, *p<0.05 *vs.* Vehicle-LF or TUDCA-HF group.

### Expression of intracellular Ca^2+^ regulatory proteins

Western blot analysis demonstrated that high fat diet feeding significantly downregulated expression of the key intracellular Ca^2+^ regulatory protein SERCA2a and phosphorylation of the negative regulator of SERCA2a - phospholamban without affecting that of Na^+^-Ca^2+^ exchanger or total phospholamban. While TUDCA did not affect the expression (total or phosphorylation) of these intracellular Ca^2+^ regulatory proteins, the ER chaperone abrogated high fat diet-induced changes in SERCA2a level and phospholamban phosphorylation without affecting Na^+^-Ca^2+^ exchanger or total phospholamban (Fig. 3F-I).

### Expression of ER stress proteins

**Figure 4:**
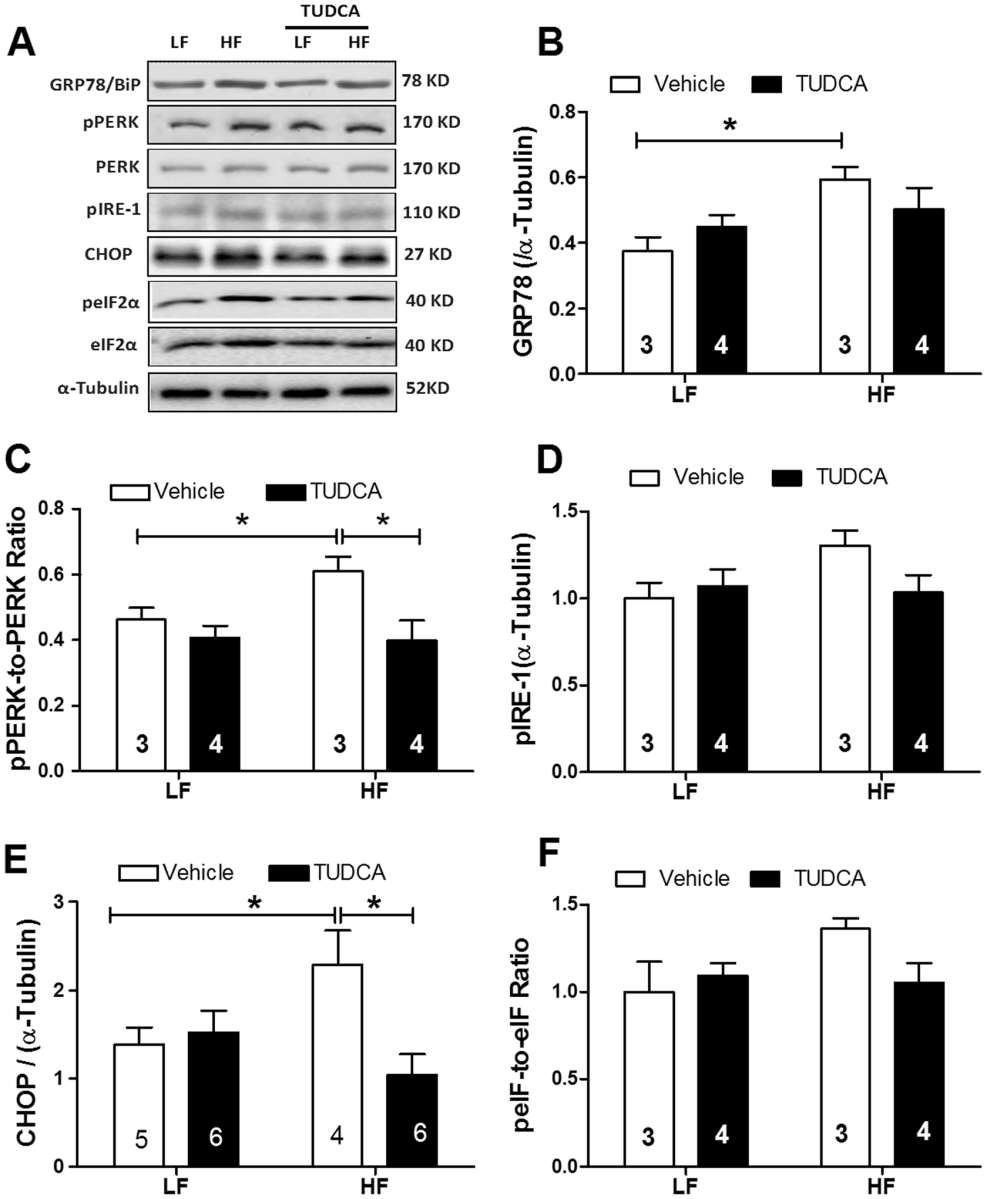
Expression of ER stress proteins in myocardium from low fat (LF) or high fat (HF)-fed C57 mice with or without TUDCA treatment (300 mg/kg for 15 days). A: Representative gel blots depicting levels of GRP78/BiP, pPERK, PERK, CHOP, eIF2α, peIF2α and α-tubulin (as loading control) using specific antibodies; B: GRP78/Bip expression; C: pPERK-to-PERK ratio; D: pIRE-1 level; E: CHOP expression; and F: peIF2α-to-eIF2α ratio. Mean±SEM; sample sizes are denoted in the bar graphs, *p<0.05 (two-way ANOVA).

Diabetes and obesity are usually associated with overt ER stress [5]. As shown in Fig. 4, high fat feeding significantly increased the levels of ER stress markers including GRP78/BiP, CHOP and phosphorylation of PERK, IRE-1 and eIF2α without affecting total protein levels of PERK and eIF2α, the effect of which was nullified by TUDCA. Treatment of the ER chaperone failed to overtly affect these ER stress markers in low fat diet-fed mouse hearts.

### Changes in cell signaling cascades of AMPK, Akt and GSK-3β

**Figure 5:**
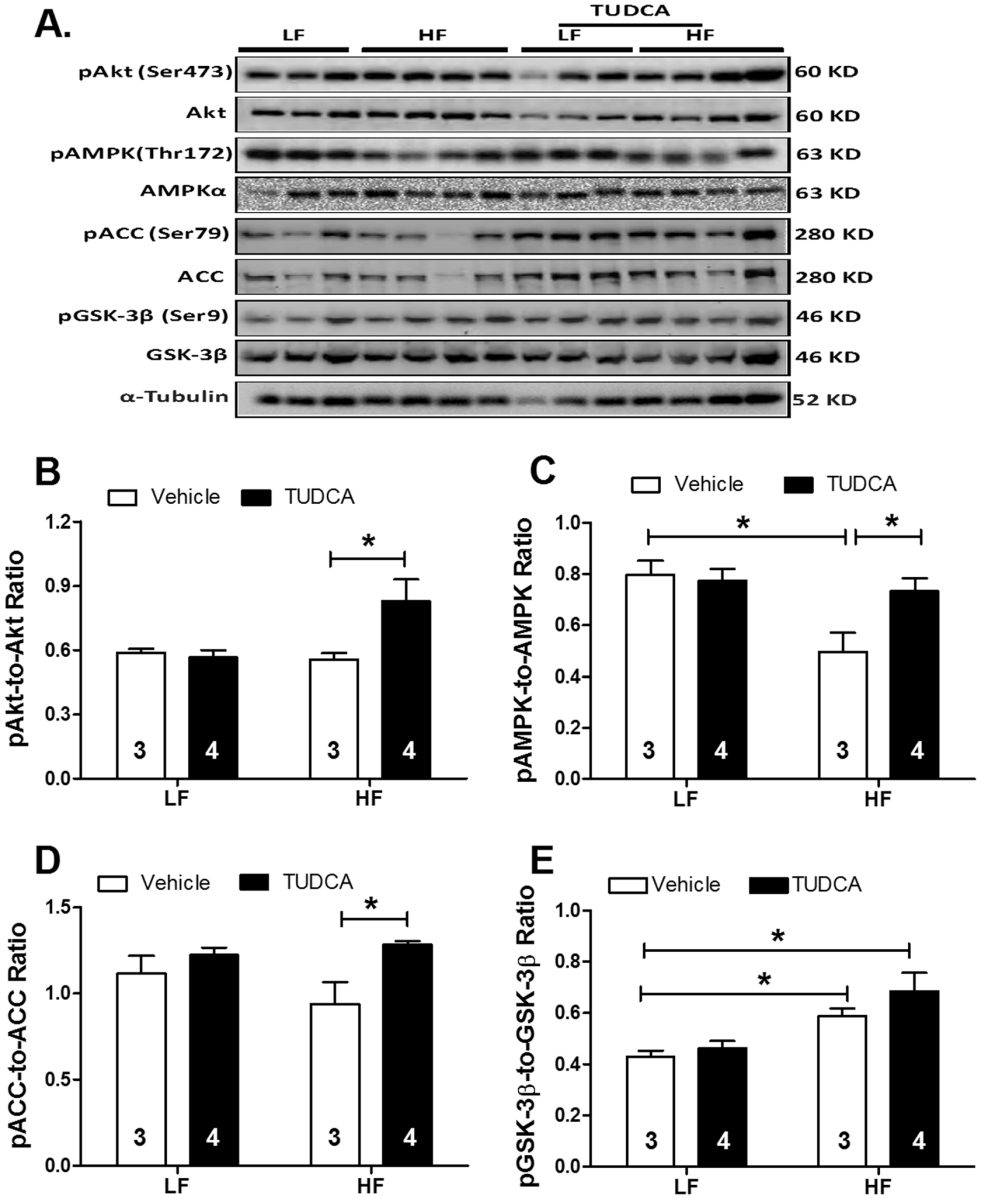
Levels of total and phosphorylated Akt, AMPK, ACC and GSK-3β in myocardium from low fat (LF) or high fat (HF)-fed C57 mice with or without TUDCA treatment (300 mg/kg for 15 days). A: Representative gel blots of Akt, pAkt, AMPK, pAMPK, ACC, pACC, GSK-3β, pGSK-3β and α-tubulin (loading control) using specific antibodies. B: pAkt-to-Akt ratio; C: pAMPK-to-AMPK ratio; D: pACC-to-ACC ratio; and E: pGSK-3β-to-GSK-3β ratio; Meanυ±SEM; sample sizes are denoted in the bar graphs; *p<0.05 (two-way ANOVA).

Given that Akt, AMPK and the Akt downstream signaling molecule GSK-3β are closely associated with regulation of cell survival and glucose homeostasis, these signaling cascades were examined in low and high fat diet-fed mice with or without TUDCA treatment. Results shown in Fig. 5 indicated that high fat diet feeding significantly suppressed activation of AMPK and enhanced GSK-3β phosphorylation without affecting the phosphorylation of Akt and ACC. TUDCA prevented high fat diet feeding-induced AMPK activation without affecting high fat diet-induced GSK-3β phosphorylation. Interestingly, TUDCA treatment elicited an increased phosphorylation of Akt and ACC in high fat diet groups. TUDCA treatment did not affect the phosphorylation of Akt, AMPK, ACC and GSK-3β in low fat group.

### Myocardial insulin signaling pathway

**Figure 6:**
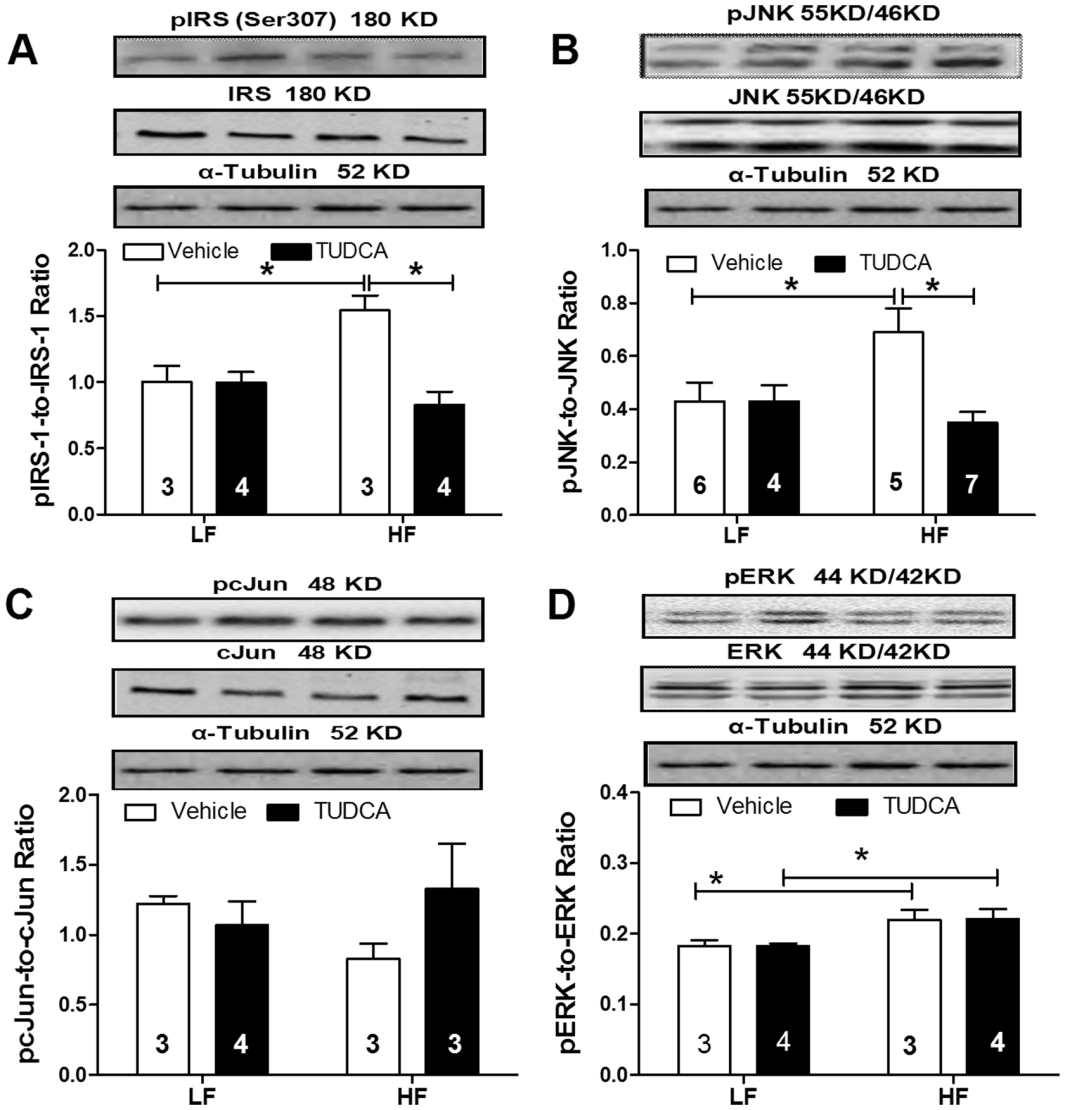
Levels of insulin signaling cascades in myocardium from low fat (LF) or high fat (HF)-fed C57 mice with or without TUDCA treatment (300 mg/kg for 15 days). A: pIRS-1-to-IRS-1 ratio; B: pJNK-to-JNK ratio; C: pcJun-to-cJun ratio; and D: pERK-to-ERK ratio. Insets: Representative gel blots of total and phosphorylated IRS-1, JNK, cJun and ERK using specific antibodies. α-tubulin was used as the loading control. Mean±SEM; sample sizes are denoted in the bar graphs; *p<0.05 (two-way ANOVA).

To examine the mechanisms involved in TUDCA-elicited cardioprotection against high fat diet feeding, phosphorylation of IRS-1, JNK c-Jun and ERK was evaluated using Western blotting. Our data revealed that high fat diet feeding significantly enhanced phosphorylation of IRS-1 (Ser^307^), JNK and ERK without affecting c-Jun phosphorylation. Although TUDCA did not affect phosphorylation of IRS-1, JNK and ERK in low fat diet feeding group, it ablated high fat diet-induced phosphorylation of IRS-1 and JNK without reversing elevated phosphorylation of ERK. Neither high fat diet feeding nor TUDCA significantly affected the levels of total IRS-1, JNK, c-Jun and ERK as well as ERK phosphorylation (Fig. 6).

## Discussion

The major findings from this study demonstrated that TUDCA treatment attenuated high fat diet- induced cardiomyocyte contractile and intracellular Ca^2+^ anomalies including depressed PS, reduced±dL/dt, and prolonged TR_90_ along with decreased ΔFFI and prolonged intracellular Ca^2+^ decay. In addition, high fat diet-induced drop in SERCA2a expression and phosphorylation of phospholamban was reversed by TUDCA. Importantly, TUDCA treatment suppressed high fat diet feeding-induced ER stress as evidenced by changes in pPERK, BiP, pIRE1 and CHOP. Furthermore, TUDCA ameliorated glucose intolerance possibly through phosphorylation of Akt and AMPK (or ACC), and suppressed phosphorylation of JNK, and IRS-1 (Ser^307^). Furthermore, TUDCA treatment ablated high fat diet-induced mitochondrial damage (evidenced by MPTP opening). These data collectively favor a unique role of the chemical chaperon TUDCA against high fat diet feeding-induced cardiomyocyte contractile and intracellular Ca^2+^ derangements, possibly through mechanisms associated with mitochondrial integrity, AMPK activation, JNK and IRS-1 serine phosphorylation.

ER reacts to pathological stimuli by upregulating ER chaperones, a response termed the unfolded protein response (UPR). However, excess ER stress or perturbation in ER function leads to apoptosis in afflicted cells [32], [33]. Obesity is known to contribute to the development of insulin resistance and diabetes through inducing ER stress in animals [5] and humans [34]. Given the pivotal role of obesity as an independent risk factor for cardiovascular diseases, targeting ER stress may be one innovative approach towards the management of cardiovascular complications associated with obesity and diabetes [35]. To this end, a plethora of studies have demonstrated that sustained ER stress may lead to pathological heart conditions such as heart failure, ischemia-reperfusion injury [36], cardiac hypertrophy [12] and atherosclerosis [18]. Previous data from our laboratory indicated that ER stress is closely associated with cardiac contractile anomalies resulted from oxidative stress [37] and sepsis [38]. More recently, finding from our laboratory demonstrated that TUDCA treatment may attenuate cardiac contractile defects in *ob/ob* mice via alleviation of ER stress [26]. Along the same line, findings from our current study revealed that TUDCA ameliorated glucose intolerance, ER stress and cardiomyocyte contractile dysfunction in fat diet-induced obesity. A unique finding from our current study was that the proapoptotic CHOP protein was effectively inhibited with TUDCA. Despite the beneficial effect of TUDCA on glucose metabolism and ER stress, the chemical chaperon was unable to counteract high fat diet-induced increase in body and heart weights, excluding the possible secondary effect resulted from body weight reduction.

SERCA2a and Na^+^-Ca^2+^ exchanger are the main machineries responsible for removing Ca^2+^ from cytosol during diastole. The reduced cardiac protein expression of SERCA2a observed in this study may underscore the diastolic dysfunction in obesity (prolongation in relengthening duration and intracellular Ca^2+^ clearing). Impaired SERCA activity has been associated with ER stress [18], [39], consistent with data from our study. Obesity contributes to insulin resistance via ER stress-induced activation of JNK, IRS-1 (Ser^307^) and the proinflammatory signaling cascades [9]. Previous data from our laboratory displayed that impaired myocardial insulin signaling may play a critical role in contractile dysfunction in obesity [26], [40]. Further study is warranted to elucidate the role of intracellular Ca^2+^ regulation in TUDCA-elicited beneficial effect against high fat diet-induced cardiomyocyte contractile and intracellular Ca^2+^ derangements.

Data from our study revealed that TUDCA restored high fat diet-induced decrease in AMPK phosphorylation. These data are consistent with our recent findings of decreased AMPK activity in hearts from mice exposed to the similar fat diet regimen [24]. AMPK is key regulator of cellular and body energy metabolism which is activated by an increase in the AMP-to-ATP ratio. AMPK increases energy supply by switching on ATP-generating pathways and decreases energy demand by switching off ATP-consuming pathways [41]. A recent report revealed that AMPK suppresses ER-associated apoptotic signaling in cardiomyocytes [17]. Likewise, AMPK activation was found to inhibit oxidized LDL-triggered ER stress via attenuation of SERCA oxidation [18]. It may be speculated that TUDCA-induced AMPK activation following high fat diet feeding helps to maintain myocardial energy fuel and glucose homeostasis in obese hearts. Moreover, TUDCA-induced inactivation of ACC (through ACC phosphorylation) may promote β-oxidation and ameliorate perturbed fatty acid oxidation following high fat diet feeding. This is supported by our recent finding where TUDCA inhibits the unsaturated fatty acid palmitic acid-induced cardiomyocyte contractile dysfunction [26]. These properties of TUDCA on myocardial energy fuel, glucose and lipid metabolism may contribute to TUDCA-offered cardioprotection independent of ER stress alleviation.

The phosphatidylinositol-3 kinase (PI3K)-Akt-GSK-3β signaling pathway is essential for cell metabolism and survival. Akt activation is implicated in insulin sensitivity and cell survival [42]. Our data demonstrated that TUDCA increased Akt activation in high fat diet- but not low fat diet-fed mouse hearts. The mechanism(s) through which TUDCA increases Akt activation is still unclear. Nevertheless, it was shown that TUDCA activates Akt in human hepatocytes via a G-protein coupled mechanism [43]. Consistently, TUDCA was found to activate Akt in skeletal muscles in obese human subjects [44]. Our data revealed that TUDCA restored high fat diet-induced mitochondrial damage as evidenced by TEM and mPTP openings. ER stress is closely associated with mitochondrial damage [22]. Mitochondrial potential (Δψ) has been implicated in the maintenance of mitochondrial integrity. It has been demonstrated NAD^+^ levels are required for normal mitochondrial fatty acid oxidation [45]. On the other hand, NAD^+^ release is used as an index of mPTP opening. In our hands, TUDCA treatment was capable of maintaining NAD^+^ levels following high fat diet. Our TEM results indicated TUDCA ameliorated the ultrastructural abnormalities induced by high fat diet, as evidenced by preserved mitochondrial integrity and density. Mitochondria are indispensable for maintaining cardiomyocyte energy metabolism, intracellular Ca^2+^ homeostasis and apoptosis. Among many regulators for mitochondria, GSK-3β inactivation through Ser9 phosphorylation is known to preserve mitochondrial function through inhibiting mPTP opening. TUDCA is capable of reducing ER stress thus to protect against mPTP opening in diabetic hearts [46]. To the contrary, the diabetes- and obesity-prone C57BL/6J mice exhibited increased GSK-3 activity [47]. Our data revealed increased GSK-3β phosphorylation in high fat diet-fed mice following TUDCA treatment, favoring a likely role of GSK-3β phosphorylation (or inactivation of GSK-3β) in the preserved mitochondrial function in high fat diet-induced obesity. This is in line with increased Akt phosphorylation in TUDCA-treated high fat diet group, as Akt is known to be upstream of GSK-3β [42]. Although it is not entirely clear at this point, enhanced GSK-3β phosphorylation following high fat feeding may reflect a compensatory mechanism of the heart to preserve mitochondrial function.

Experimental limitations: Although our current study has provided several lines of causal relationship among ER stress, TUDCA treatment and myocardial function in high fat diet-induced obesity, it does not offer any conclusive answer with regards to the precise role of ER stress in human obesity-associated heart dysfunction. For the assessment of mPTP opening, use of NAD levels is non-specific and may be affected by the presence of enzymes (such as alcohol metabolizing enzymes) requiring NAD as a coenzyme. Another limitation of our study is the assessment of cardiomyocyte contractile function in a sub-physiological setting. Use of high stimulus frequency close to the physiological setting at 5 Hz cannot fully reveal some of the impaired contractile parameters (e.g., prolongation of TR_90_ seen at lower stimulus frequencies may be masked by the narrowed cardiac cycle duration).

In summary, data from our present study revealed that the chemical chaperon TUDCA may ameliorate high fat diet-induced cardiomyocyte contractile and intracellular Ca^2+^ anomalies. The cardioprotective effects of the ER chaperone may be associated with improved insulin and AMPK signaling, alleviated phosphorylation of the negative regulators for insulin signaling including JNK and IRS-1 (serine) and preserved mitochondrial integrity. These findings should shed some lights on the therapeutic implication of chemical chaperons in the management of pathological conditions where ER stress is abundant.

## References

[1] Centers for Disease Control and Prevention (2011) National diabetes fact sheet: national estimates and general information on diabetes and prediabetes in the United States, 2011.

[2] Ren J, Kelley RO (2009) Cardiac health in women with metabolic syndrome: clinical aspects and pathophysiology. Obesity (Silver Spring) 17: 1114–1123. 19214173 10.1038/oby.2009.8

[3] Sullivan PW, Ghushchyan V, Ben-Joseph H (2008) The Effect of Obesity and Cardiometabolic Risk Factors on Expenditures and Productivity in the United States. Obesity (Silver Spring). 10.1038/oby.2008.325 19186336

[4] Zalesin KC, Franklin BA, Miller WM, Peterson ED, McCullough PA (2008) Impact of obesity on cardiovascular disease. Endocrinol Metab Clin North Am 37: 663–684. 18775358 10.1016/j.ecl.2008.06.004

[5] Ozcan U, Cao Q, Yilmaz E, Lee AH, Iwakoshi NN, et al (2004) Endoplasmic reticulum stress links obesity, insulin action, and type 2 diabetes. Science 306: 457–461. 15486293 10.1126/science.1103160

[6] Yoshida H (2007) ER stress and diseases. FEBS J 274: 630–658. 17288551 10.1111/j.1742-4658.2007.05639.x

[7] Eizirik DL, Cardozo AK, Cnop M (2008) The role for endoplasmic reticulum stress in diabetes mellitus. Endocr Rev 29: 42–61. 18048764 10.1210/er.2007-0015

[8] Kim DS, Jeong SK, Kim HR, Kim DS, Chae SW, et al (2007) Effects of triglyceride on ER stress and insulin resistance. Biochem Biophys Res Commun 363: 140–145. 17868644 10.1016/j.bbrc.2007.08.151

[9] Ozcan U, Yilmaz E, Ozcan L, Furuhashi M, Vaillancourt E, et al (2006) Chemical chaperones reduce ER stress and restore glucose homeostasis in a mouse model of type 2 diabetes. Science 313: 1137–1140. 16931765 10.1126/science.1128294 PMC4741373

[10] Groenendyk J, Sreenivasaiah PK, Kim DH, Agellon LB, Michalak M (2010) Biology of endoplasmic reticulum stress in the heart. Circ Res 107: 1185–1197. 21071716 10.1161/CIRCRESAHA.110.227033

[11] Minamino T, Kitakaze M (2010) ER stress in cardiovascular disease. J Mol Cell Cardiol 48: 1105–1110. 19913545 10.1016/j.yjmcc.2009.10.026

[12] Okada K, Minamino T, Tsukamoto Y, Liao Y, Tsukamoto O, et al (2004) Prolonged endoplasmic reticulum stress in hypertrophic and failing heart after aortic constriction: possible contribution of endoplasmic reticulum stress to cardiac myocyte apoptosis. Circulation 110: 705–712. 15289376 10.1161/01.CIR.0000137836.95625.D4

[13] Fu HY, Minamino T, Tsukamoto O, Sawada T, Asai M, et al (2008) Overexpression of endoplasmic reticulum-resident chaperone attenuates cardiomyocyte death induced by proteasome inhibition. Cardiovasc Res 79: 600–610. 18508854 10.1093/cvr/cvn128

[14] Nickson P, Toth A, Erhardt P (2007) PUMA is critical for neonatal cardiomyocyte apoptosis induced by endoplasmic reticulum stress. Cardiovasc Res 73: 48–56. 17107669 10.1016/j.cardiores.2006.10.001 PMC1832123

[15] Szegezdi E, Duffy A, O′Mahoney ME, Logue SE, Mylotte LA, et al (2006) ER stress contributes to ischemia-induced cardiomyocyte apoptosis. Biochem Biophys Res Commun 349: 1406–1411. 16979584 10.1016/j.bbrc.2006.09.009

[16] Liu XH, Zhang ZY, Sun S, Wu XD (2008) Ischemic postconditioning protects myocardium from ischemia/reperfusion injury through attenuating endoplasmic reticulum stress. Shock 30: 422–427. 18323739 10.1097/SHK.0b013e318164ca29

[17] Terai K, Hiramoto Y, Masaki M, Sugiyama S, Kuroda T, et al (2005) AMP-activated protein kinase protects cardiomyocytes against hypoxic injury through attenuation of endoplasmic reticulum stress. Mol Cell Biol 25: 9554–9575. 16227605 10.1128/MCB.25.21.9554-9575.2005 PMC1265833

[18] Dong Y, Zhang M, Wang S, Liang B, Zhao Z, et al (2010) Activation of AMP-activated protein kinase inhibits oxidized LDL-triggered endoplasmic reticulum stress in vivo. Diabetes 59: 1386–1396. 20299472 10.2337/db09-1637 PMC2874699

[19] Chen Y, Liu CP, Xu KF, Mao XD, Lu YB, et al (2008) Effect of Taurine-Conjugated Ursodeoxycholic Acid on Endoplasmic Reticulum Stress and Apoptosis Induced by Advanced Glycation End Products in Cultured Mouse Podocytes. Am J Nephrol 28: 1014–1022. 18648192 10.1159/000148209

[20] Sohn J, Khaoustov VI, Xie Q, Chung CC, Krishnan B, et al (2003) The effect of ursodeoxycholic acid on the survivin in thapsigargin-induced apoptosis. Cancer Lett 191: 83–92. 12609713 10.1016/s0304-3835(02)00624-9

[21] Ikegami T, Matsuzaki Y (2008) Ursodeoxycholic acid: Mechanism of action and novel clinical applications. Hepatol Res 38: 123–131. 18034825 10.1111/j.1872-034X.2007.00297.x

[22] Xie Q, Khaoustov VI, Chung CC, Sohn J, Krishnan B, et al (2002) Effect of tauroursodeoxycholic acid on endoplasmic reticulum stress-induced caspase-12 activation. Hepatology 36: 592–601. 12198651 10.1053/jhep.2002.35441

[23] Azfer A, Niu J, Rogers LM, Adamski FM, Kolattukudy PE (2006) Activation of endoplasmic reticulum stress response during the development of ischemic heart disease. Am J Physiol Heart Circ Physiol 291: H1411–H1420. 16617122 10.1152/ajpheart.01378.2005 PMC1575464

[24] Turdi S, Kandadi MR, Zhao J, Huff AF, Du M, et al (2011) Deficiency in AMP-activated protein kinase exaggerates high fat diet-induced cardiac hypertrophy and contractile dysfunction. J Mol Cell Cardiol 50: 712–722. 21167835 10.1016/j.yjmcc.2010.12.007 PMC3049828

[25] Wilson CR, Tran MK, Salazar KL, Young ME, Taegtmeyer H (2007) Western diet, but not high fat diet, causes derangements of fatty acid metabolism and contractile dysfunction in the heart of Wistar rats. Biochem J 406: 457–467 BJ20070392 [pii];10.1042/BJ20070392 [doi]. 17550347 10.1042/BJ20070392 PMC2049036

[26] Ceylan-Isik AF, Sreejayan N, Ren J (2011) Endoplasmic reticulum chaperon tauroursodeoxycholic acid alleviates obesity-induced myocardial contractile dysfunction. J Mol Cell Cardiol 50: 107–116. 21035453 10.1016/j.yjmcc.2010.10.023 PMC3018539

[27] Hintz KK, Aberle NS, Ren J (2003) Insulin resistance induces hyperleptinemia, cardiac contractile dysfunction but not cardiac leptin resistance in ventricular myocytes. Int J Obes Relat Metab Disord 27: 1196–1203. 14513067 10.1038/sj.ijo.0802389

[28] Dong F, Li Q, Sreejayan N, Nunn JM, Ren J (2007) Metallothionein prevents high-fat diet induced cardiac contractile dysfunction: role of peroxisome proliferator activated receptor gamma coactivator 1alpha and mitochondrial biogenesis. Diabetes 56: 2201–2212. 17575086 10.2337/db06-1596

[29] Oka S, Alcendor R, Zhai P, Park JY, Shao D, et al (2011) PPARalpha-Sirt1 complex mediates cardiac hypertrophy and failure through suppression of the ERR transcriptional pathway. Cell Metab 14: 598–611. 22055503 10.1016/j.cmet.2011.10.001 PMC3217210

[30] Zhu J, Rebecchi MJ, Glass PSA, Brink PR, Liu L (2011) Cardioprotection of the aged rat heart by GSK-3 inhibitor is attenuated: age-related changes in mitochondrial permeability transition pore modulation. American Journal of Physiology - Heart and Circulatory Physiology 300: H922–H930. 21217064 10.1152/ajpheart.00860.2010

[31] Ouwens DM, Diamant M, Fodor M, Habets DD, Pelsers MM, et al (2007) Cardiac contractile dysfunction in insulin-resistant rats fed a high-fat diet is associated with elevated CD36-mediated fatty acid uptake and esterification. Diabetologia 50: 1938–1948. 17639306 10.1007/s00125-007-0735-8 PMC2039861

[32] Engin F, Hotamisligil GS (2010) Restoring endoplasmic reticulum function by chemical chaperones: an emerging therapeutic approach for metabolic diseases. Diabetes Obes Metab 12 Suppl 2108–115. 21029307 10.1111/j.1463-1326.2010.01282.x

[33] Xu C, Bailly-Maitre B, Reed JC (2005) Endoplasmic reticulum stress: cell life and death decisions. J Clin Invest 115: 2656–2664. 16200199 10.1172/JCI26373 PMC1236697

[34] Boden G, Duan X, Homko C, Molina EJ, Song W, et al (2008) Increase in endoplasmic reticulum stress-related proteins and genes in adipose tissue of obese, insulin-resistant individuals. Diabetes 57: 2438–2444. 18567819 10.2337/db08-0604 PMC2518495

[35] Toth A, Nickson P, Mandl A, Bannister ML, Toth K, et al (2007) Endoplasmic reticulum stress as a novel therapeutic target in heart diseases. Cardiovasc Hematol Disord Drug Targets 7: 205–218. 17896961 10.2174/187152907781745260

[36] Martindale JJ, Fernandez R, Thuerauf D, Whittaker R, Gude N, et al (2006) Endoplasmic reticulum stress gene induction and protection from ischemia/reperfusion injury in the hearts of transgenic mice with a tamoxifen-regulated form of ATF6. Circ Res 98: 1186–1193. 16601230 10.1161/01.RES.0000220643.65941.8d

[37] Guo R, Ma H, Gao F, Zhong L, Ren J (2009) Metallothionein alleviates oxidative stress-induced endoplasmic reticulum stress and myocardial dysfunction. J Mol Cell Cardiol 47: 228–237. 19344729 10.1016/j.yjmcc.2009.03.018 PMC2703679

[38] Ceylan-Isik AF, Zhao P, Zhang B, Xiao X, Su G, et al (2010) Cardiac overexpression of metallothionein rescues cardiac contractile dysfunction and endoplasmic reticulum stress but not autophagy in sepsis. J Mol Cell Cardiol 48: 367–378. 19914257 10.1016/j.yjmcc.2009.11.003 PMC2813369

[39] Cardozo AK, Ortis F, Storling J, Feng YM, Rasschaert J, et al (2005) Cytokines downregulate the sarcoendoplasmic reticulum pump Ca2+ ATPase 2b and deplete endoplasmic reticulum Ca2+, leading to induction of endoplasmic reticulum stress in pancreatic beta-cells. Diabetes 54: 452–461. 15677503 10.2337/diabetes.54.2.452

[40] Dong F, Ren J (2009) Adiponectin improves cardiomyocyte contractile function in db/db diabetic obese mice. Obesity (Silver Spring) 17: 262–268. 19057532 10.1038/oby.2008.545

[41] Hardie DG (2004) AMP-activated protein kinase: the guardian of cardiac energy status. J Clin Invest 114: 465–468. 15314681 10.1172/JCI22683 PMC503780

[42] Sussman MA, Volkers M, Fischer K, Bailey B, Cottage CT, et al (2011) Myocardial AKT: The Omnipresent Nexus. Physiol Rev 91: 1023–1070. 21742795 10.1152/physrev.00024.2010 PMC3674828

[43] Fang Y, Studer E, Mitchell C, Grant S, Pandak WM, et al (2007) Conjugated bile acids regulate hepatocyte glycogen synthase activity in vitro and in vivo via Galphai signaling. Mol Pharmacol 71: 1122–1128. 17200418 10.1124/mol.106.032060

[44] Kars M, Yang L, Gregor MF, Mohammed BS, Pietka TA, et al (2010) Tauroursodeoxycholic Acid may improve liver and muscle but not adipose tissue insulin sensitivity in obese men and women. Diabetes 59: 1899–1905. 20522594 10.2337/db10-0308 PMC2911053

[45] Hwang JH, Kim DW, Jo EJ, Kim YK, Jo YS, et al (2009) Pharmacological stimulation of NADH oxidation ameliorates obesity and related phenotypes in mice. Diabetes 58: 965–974. 19136651 10.2337/db08-1183 PMC2661596

[46] Miki T, Miura T, Hotta H, Tanno M, Yano T, et al (2009) Endoplasmic reticulum stress in diabetic hearts abolishes erythropoietin-induced myocardial protection by impairment of phospho-glycogen synthase kinase-3beta-mediated suppression of mitochondrial permeability transition. Diabetes 58: 2863–2872. 19755525 10.2337/db09-0158 PMC2780889

[47] Eldar-Finkelman H, Schreyer SA, Shinohara MM, LeBoeuf RC, Krebs EG (1999) Increased glycogen synthase kinase-3 activity in diabetes- and obesity-prone C57BL/6J mice. Diabetes 48: 1662–1666. 10426388 10.2337/diabetes.48.8.1662

