# Correction: Tauroursodeoxycholic Acid Mitigates High Fat Diet-Induced Cardiomyocyte Contractile and Intracellular Ca^2+^ Anomalies

**DOI:** 10.1371/journal.pone.0154907

**Published:** 2016-04-28

**Authors:** Subat Turdi, Nan Hu, Jun Ren

The authors would like to correct Fig 6. In preparation of the figure for publication, the authors ran a representative gel for each of the experimental samples shown in panels A, B, C, and D of Fig 6. An α-Tubulin 52 KD loading control was then separately run and used as the representative loading control for all panels in Fig 6.

**Fig 6:**
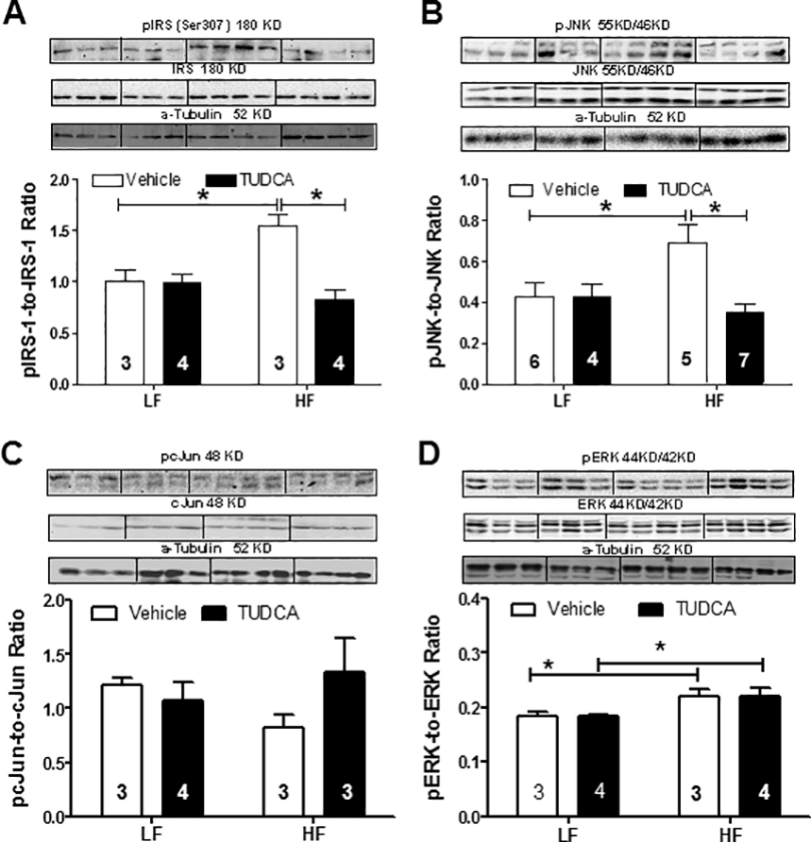
Levels of insulin signaling cascades in myocardium from low fat (LF) or high fat (HF)-fed C57 mice with or without TUDCA treatment (300 mg/kg for 15 days). A: pIRS-1-to-IRS-1 ratio; B: pJNK-to-JNK ratio; C: pcJun-to-cJun ratio; and D: pERK-to-ERK ratio. Insets: Representative gel blots of total and phosphorylated IRS-1, JNK, cJun and ERK using specific antibodies. α-tubulin was used as the loading control. Mean±SEM; sample sizes are denoted in the bar graphs; *p<0.05 (two-way ANOVA).

The authors have provided a corrected version of Fig 6 here. The corrected Fig 6 shows the original, whole gels and their matching loading controls. Vertical black lines denote a rearrangement of bands from the raw gels. The authors confirm that these changes do not alter their findings and have provided raw, uncropped blots as Supporting Information.

## Supporting Information

S1 File Raw blots used to create the corrected version of Fig 6. (PPT)
