# Supplementary material for: Correction: Tauroursodeoxycholic Acid Mitigates High Fat Diet-Induced Cardiomyocyte Contractile and Intracellular Ca2+ Anomalies
Source: PLoS One. 2016 Apr 28;11(4):e0154907. doi: 10.1371/journal.pone.0154907 (PMC4849677; doi:10.1371/journal.pone.0154907)
Supplement: S1 File — (PPT) [file pone.0154907.s001.ppt]

## Slide 1
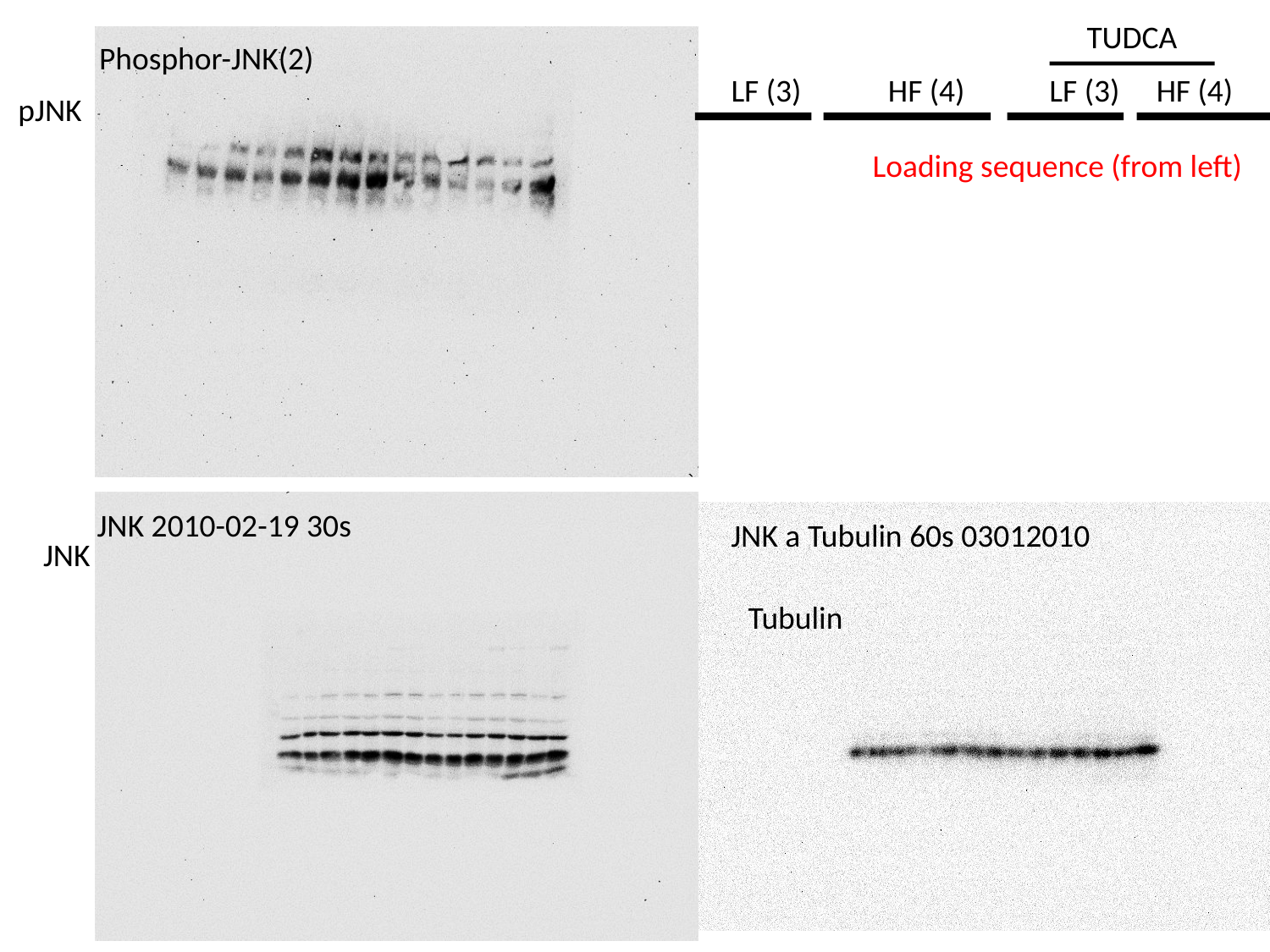

TUDCA
Phosphor-JNK(2)
LF (3)
HF (4)
LF (3)
HF (4)
pJNK
Loading sequence (from left)
JNK 2010-02-19 30s
JNK a Tubulin 60s 03012010
JNK
Tubulin

## Slide 2
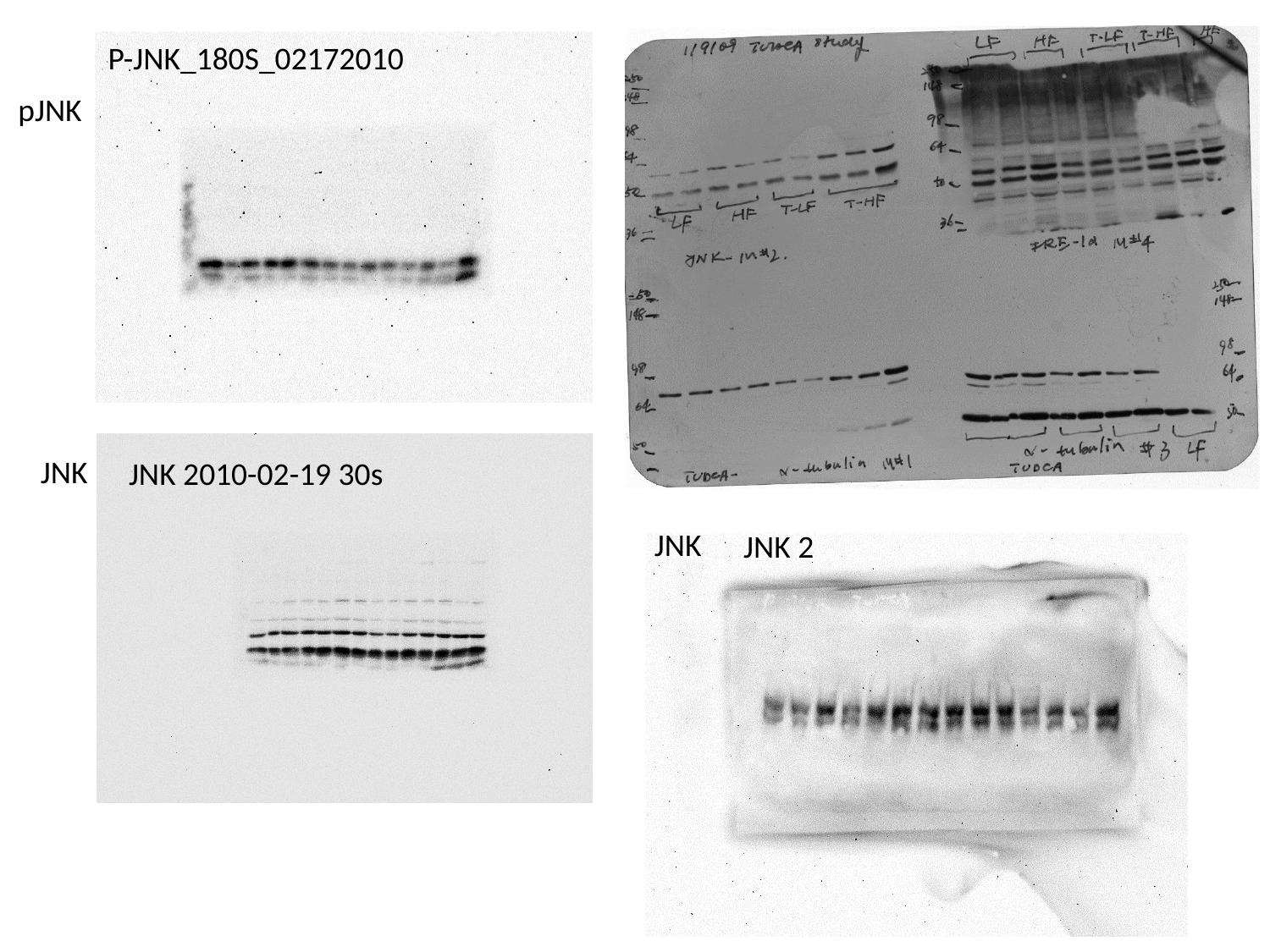

P-JNK_180S_02172010
pJNK
JNK
JNK 2010-02-19 30s
JNK
JNK 2

## Slide 3
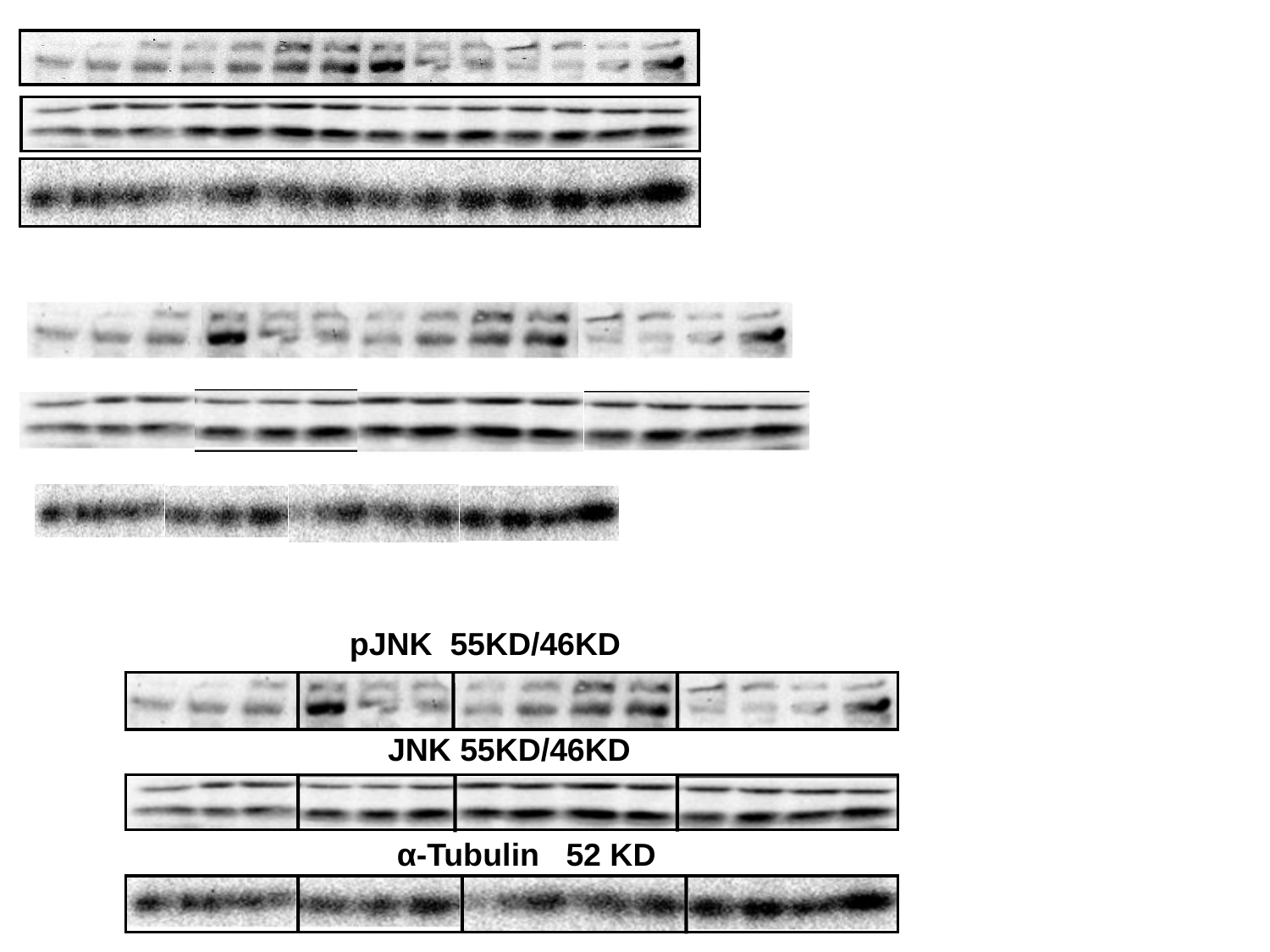

pJNK 55KD/46KD
JNK 55KD/46KD
 α-Tubulin 52 KD

## Slide 4
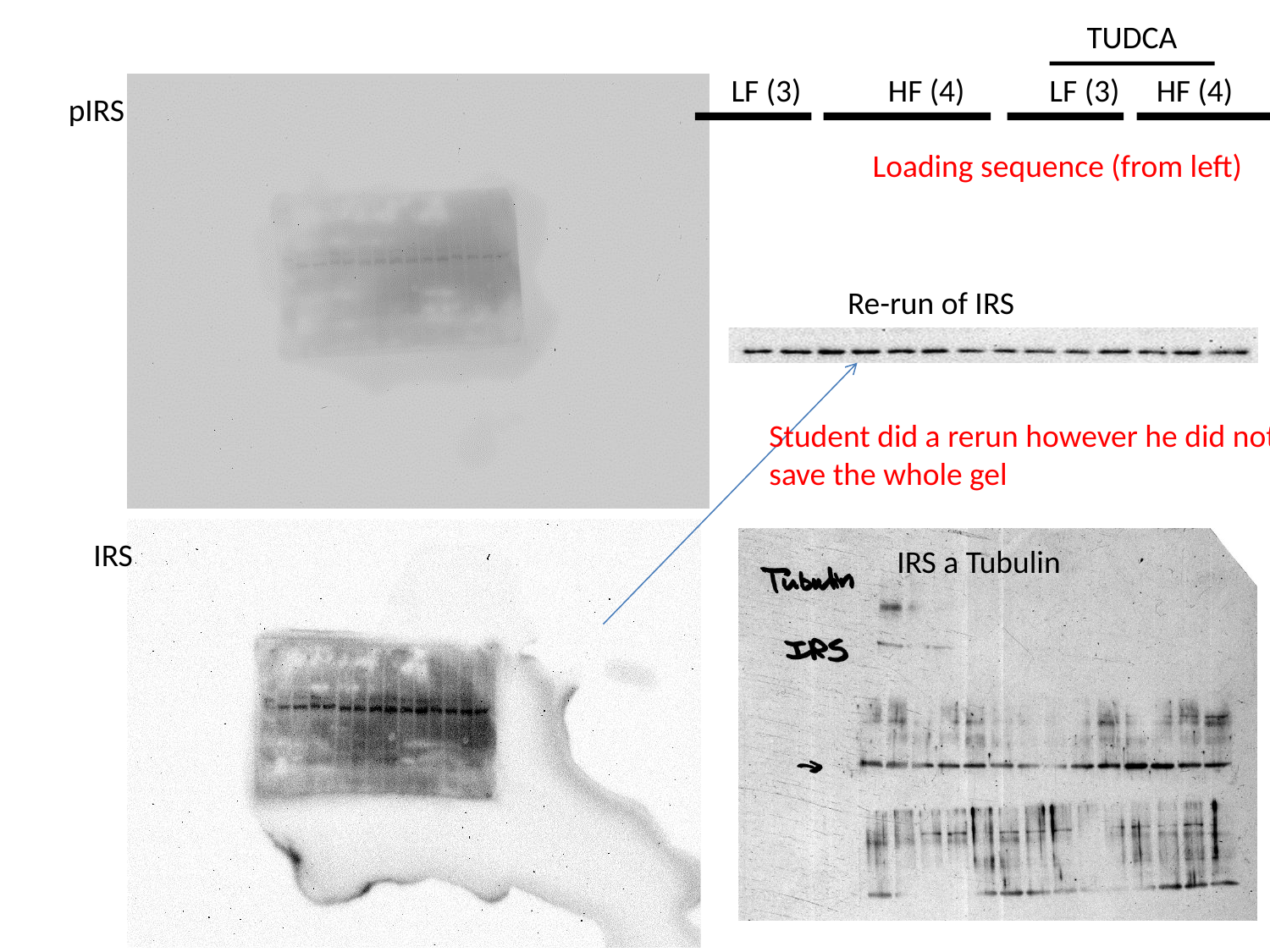

TUDCA
LF (3)
HF (4)
LF (3)
HF (4)
pIRS
Loading sequence (from left)
Re-run of IRS
Student did a rerun however he did not
save the whole gel
IRS
IRS a Tubulin

## Slide 5
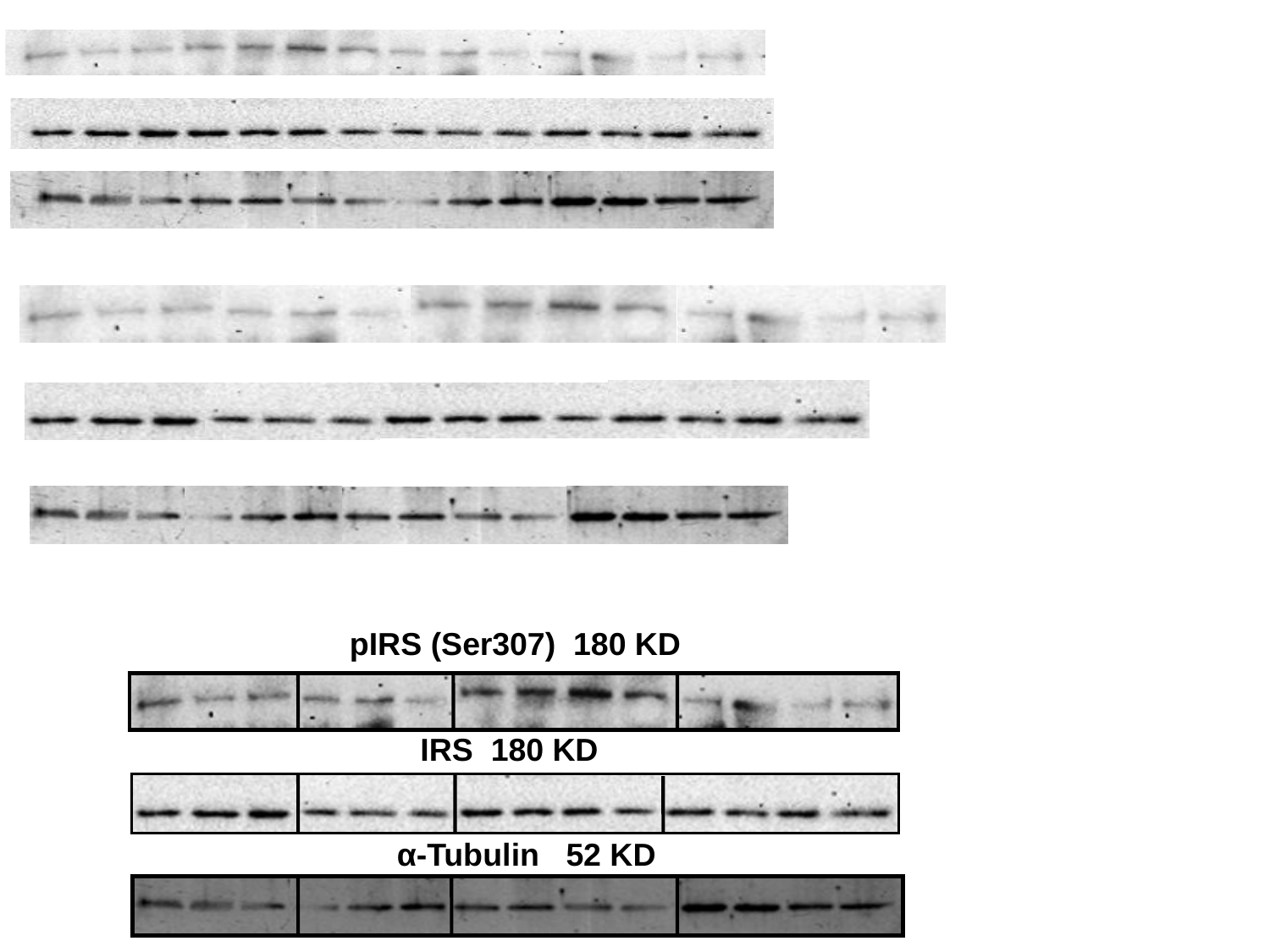

pIRS (Ser307) 180 KD
IRS 180 KD
 α-Tubulin 52 KD

## Slide 6
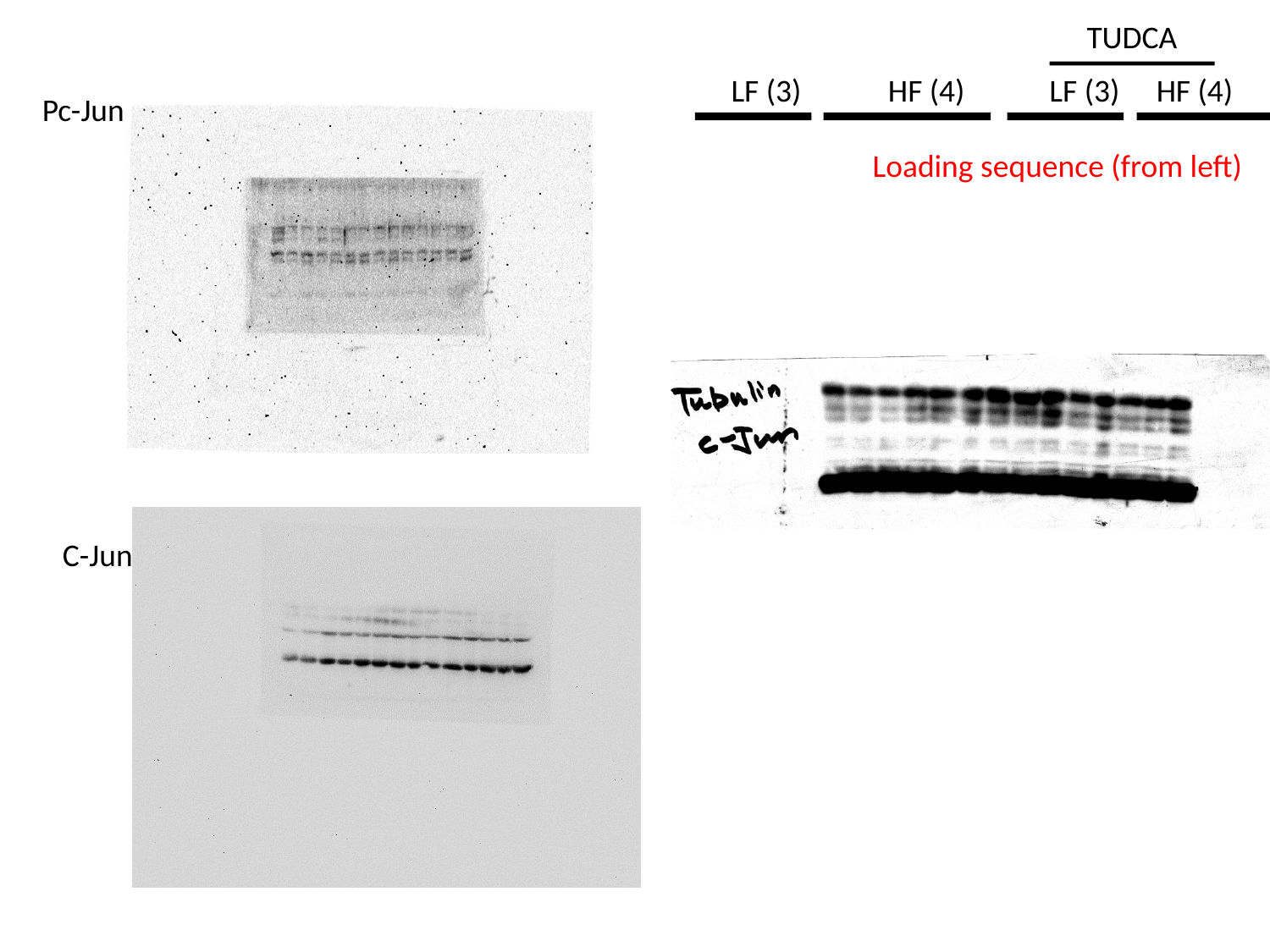

TUDCA
LF (3)
HF (4)
LF (3)
HF (4)
Pc-Jun
Loading sequence (from left)
C-Jun

## Slide 7
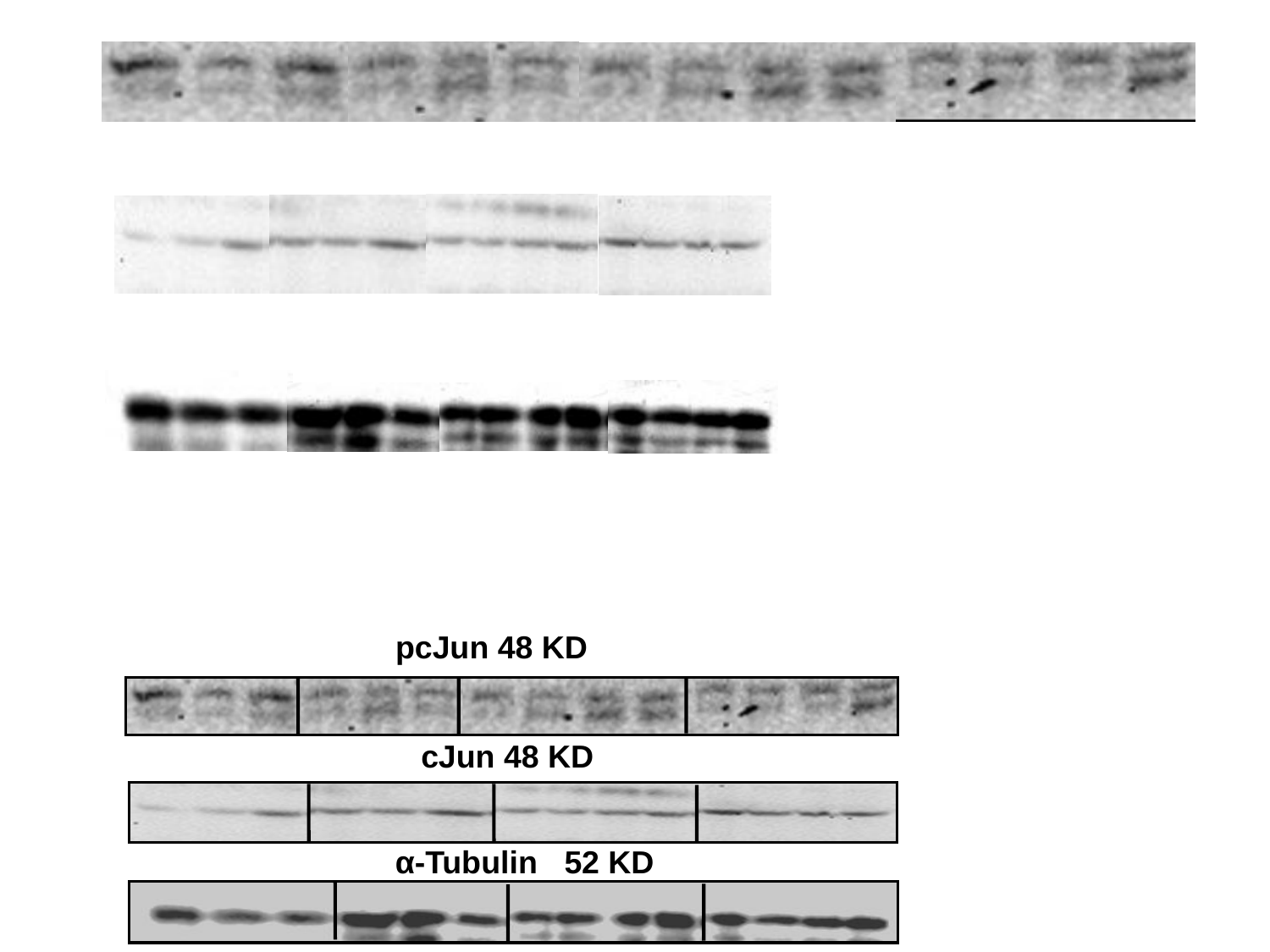

pcJun 48 KD
cJun 48 KD
 α-Tubulin 52 KD

## Slide 8
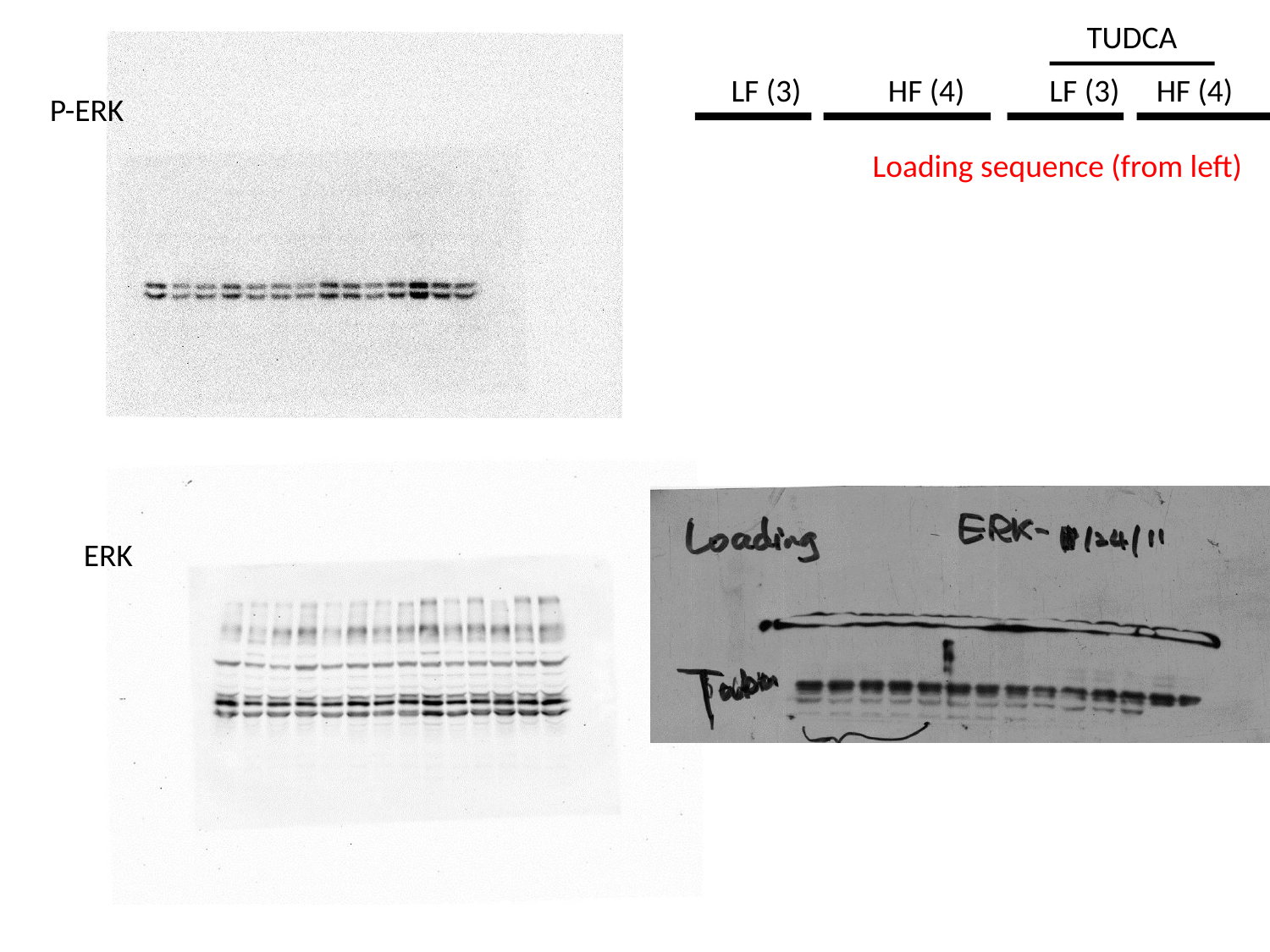

TUDCA
LF (3)
HF (4)
LF (3)
HF (4)
P-ERK
Loading sequence (from left)
ERK

## Slide 9
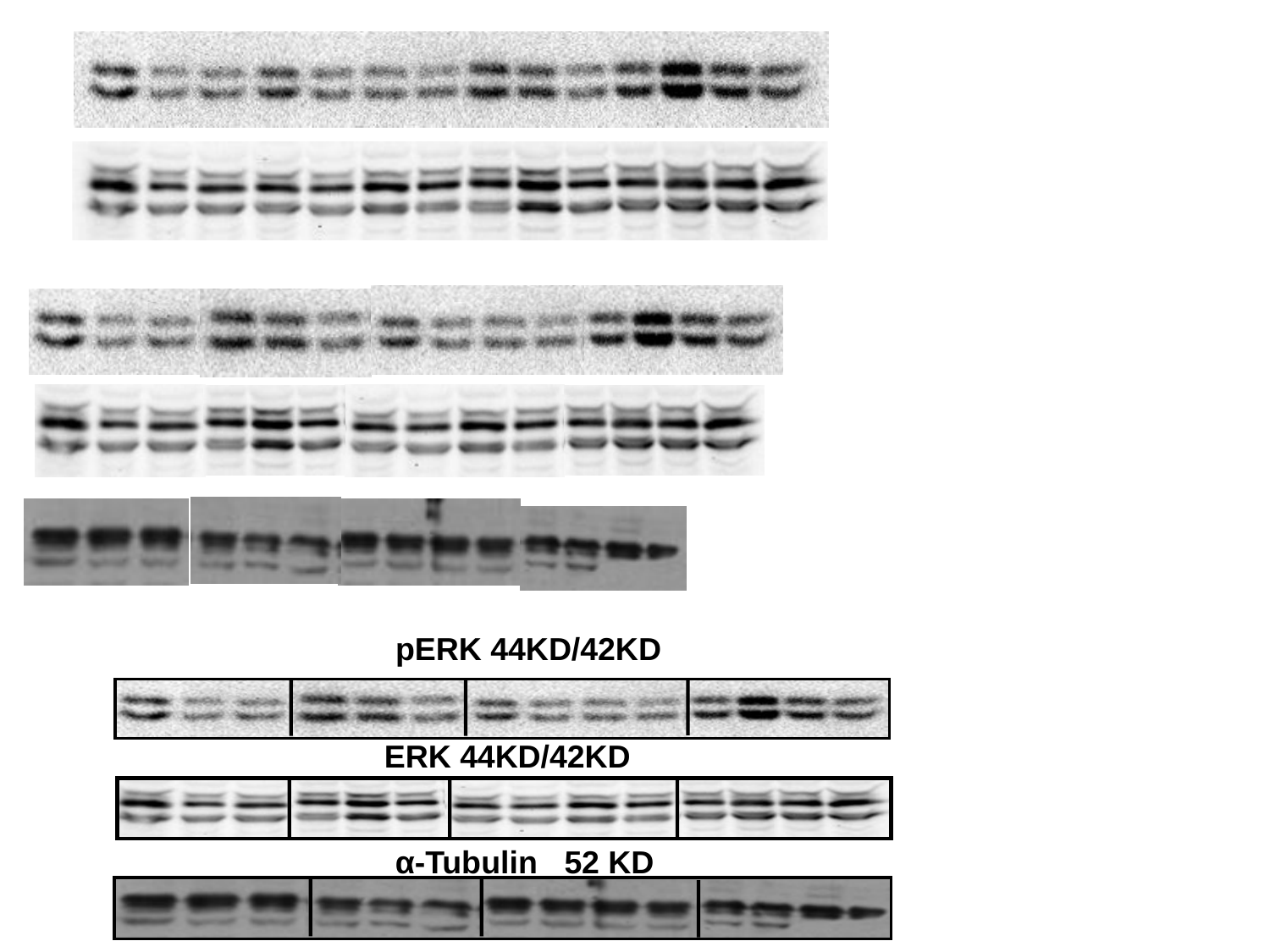

pERK 44KD/42KD
ERK 44KD/42KD
 α-Tubulin 52 KD
